# Causal association between snoring and stroke: a Mendelian randomization study in a Chinese population

**DOI:** 10.1016/j.lanwpc.2023.101001

**Published:** 2024-01-23

**Authors:** Yunqing Zhu, Zhenhuang Zhuang, Jun Lv, Dianjianyi Sun, Pei Pei, Ling Yang, Iona Y. Millwood, Robin G. Walters, Yiping Chen, Huaidong Du, Xianping Wu, Dan Schmidt, Daniel Avery, Junshi Chen, Zhengming Chen, Liming Li, Canqing Yu, China Kadoorie Biobank Collaborative Group, Junshi Chen, Zhengming Chen, Robert Clarke, Rory Collins, Yu Guo, Liming Li, Jun Lv, Richard Peto, Robin Walter, Daniel Avery, Derrick Bennett, Ruth Boxall, Sue Burgess, Ka Hung Chan, Yumei Chang, Yiping Chen, Zhengming Chen, Johnathan Clarke, Robert Clarke, Huaidong Du, Ahmed Edris Mohamed, Zammy Fairhurst-Hunter, Hannah Fry, Mike Hill, Michael Holmes, Pek Kei Im, Andri Iona, Maria Kakkoura, Christiana Kartsonaki, Rene Kerosi, Kuang Lin, Mohsen Mazidi, Iona Millwood, Sam Morris, Qunhua Nie, Alfred Pozarickij, Paul Ryder, Saredo Said, Dan Schmidt, Paul Sherliker, Becky Stevens, Iain Turnbull, Robin Walters, Lin Wang, Neil Wright, Ling Yang, Xiaoming Yang, Pang Yao, Yu Guo, Xiao Han, Can Hou, Jun Lv, Pei Pei, Chao Liu, Canqing Yu, Qingmei Xia, Zengchang Pang, Ruqin Gao, Shanpeng Li, Haiping Duan, Shaojie Wang, Yongmei Liu, Ranran Du, Yajing Zang, Liang Cheng, Xiaocao Tian, Hua Zhang, Yaoming Zhai, Feng Ning, Xiaohui Sun, Feifei Li, Silu Lv, Junzheng Wang, Wei Hou, Wei Sun, Shichun Yan, Xiaoming Cui, Chi Wang, Zhenyuan Wu, Yanjie Li, Quan Kang, Huiming Luo, Tingting Ou, Xiangyang Zheng, Zhendong Guo, Shukuan Wu, Yilei Li, Huimei Li, Ming Wu, Yonglin Zhou, Jinyi Zhou, Ran Tao, Jie Yang, Jian Su, Fang Liu, Jun Zhang, Yihe Hu, Yan Lu, Liangcai Ma, Aiyu Tang, Shuo Zhang, Jianrong Jin, Jingchao Liu, Mei Lin, Zhenzhen Lu, Lifang Zhou, Changping Xie, Jian Lan, Tingping Zhu, Yun Liu, Liuping Wei, Liyuan Zhou, Ningyu Chen, Yulu Qin, Sisi Wang, Xianping Wu, Ningmei Zhang, Xiaofang Chen, Xiaoyu Chang, Mingqiang Yuan, Xia Wu, Xiaofang Chen, Wei Jiang, Jiaqiu Liu, Qiang Sun, Faqing Chen, Xiaolan Ren, Caixia Dong, Hui Zhang, Enke Mao, Xiaoping Wang, Tao Wang, Xi zhang, Kai Kang, Shixian Feng, Huizi Tian, Lei Fan, XiaoLin Li, Huarong Sun, Pan He, Xukui Zhang, Min Yu, Ruying Hu, Hao Wang, Xiaoyi Zhang, Yuan Cao, Kaixu Xie, Lingli Chen, Dun Shen, Xiaojun Li, Donghui Jin, Li Yin, Huilin Liu, Zhongxi Fu, Xin Xu, Hao Zhang, Jianwei Chen, Yuan Peng, Libo Zhang, Chan Qu

**Affiliations:** a Department of Epidemiology & Biostatistics, School of Public Health, Peking University, Beijing, 100191, China; b Peking University Center for Public Health and Epidemic Preparedness & Response, Beijing, 100191, China; c Key Laboratory of Epidemiology of Major Diseases (Peking University), Ministry of Education, Beijing, 100191, China; d Medical Research Council Population Health Research Unit at the University of Oxford, Oxford, OX3 7LF, United Kingdom; e Clinical Trial Service Unit & Epidemiological Studies Unit (CTSU), Nuffield Department of Population Health, University of Oxford, Oxford, OX3 7LF, United Kingdom; f Suzhou Centers for Disease Control, NO.72 Sanxiang Road, Gusu District, Suzhou, 215004, Jiangsu, China; g China National Center for Food Safety Risk Assessment, Beijing, 100022, China

**Keywords:** Snoring, Stroke, Body mass index, Mendelian randomization

## Abstract

**Background:**

Previous observational studies established a positive relationship between snoring and stroke. We aimed to investigate the causal effect of snoring on stroke.

**Methods:**

Based on 82,339 unrelated individuals with qualified genotyping data of Asian descent from the China Kadoorie Biobank (CKB), we conducted a Mendelian randomization (MR) analysis of snoring and stroke. Genetic variants identified in the genome-wide association analysis (GWAS) of snoring in CKB and UK Biobank (UKB) were selected for constructing genetic risk scores (GRS). A two-stage method was applied to estimate the associations of the genetically predicted snoring with stroke and its subtypes. Besides, MR analysis among the non-obese group (body mass index, BMI <24.0 kg/m^2^), as well as multivariable MR (MVMR), were performed to control for potential pleiotropy from BMI. In addition, the inverse-variance weighted (IVW) method was applied to estimate the causal association with genetic variants identified in CKB GWAS.

**Findings:**

Positive associations were found between snoring and total stroke, hemorrhagic stroke (HS), and ischemic stroke (IS). With GRS of CKB, the corresponding HRs (95% CIs) were 1.56 (1.15, 2.12), 1.50 (0.84, 2.69), 2.02 (1.36, 3.01), and the corresponding HRs (95% CIs) using GRS of UKB were 1.78 (1.30, 2.43), 1.94 (1.07, 3.52), and 1.74 (1.16, 2.61). The associations remained stable in the MR among the non-obese group, MVMR analysis, and MR analysis using the IVW method.

**Interpretation:**

This study suggests that, among Chinese adults, genetically predicted snoring could increase the risk of total stroke, IS, and HS, and the causal effect was independent of BMI.

**Funding:**

National Natural Science Foundation of China, 10.13039/501100017647Kadoorie Charitable Foundation Hong Kong, UK Wellcome Trust, 10.13039/501100012166National Key R&D Program of China, Chinese Ministry of Science and Technology.

## Graphical abstract

**Figure:**
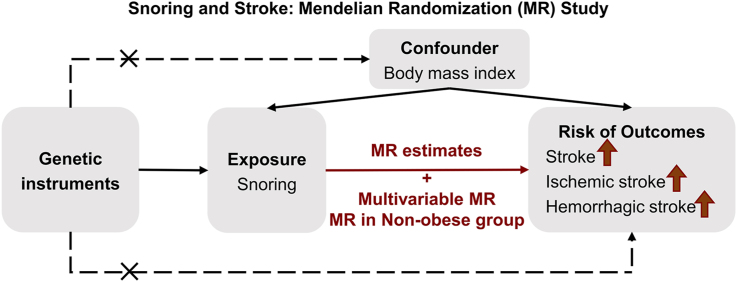

**Research in context**

**Evidence before this study**

We searched PubMed and Google Scholar for articles published before May 30, 2023, using combined terms of snoring and stroke. No restrictions were applied to the study type or language. The relevant studies were also found by checking the reference lists of identified articles. Previous observational studies estimated the relationship between snoring and stroke, and they didn't reach a consistent conclusion. Mendelian randomization studies evaluating their association were conducted in the European population, the casual association among the Chinese was unclear.

**Added value of this study**

This is the first study to investigate the causal relationship between snoring and stroke among the Asian population. Based on 82,339 unrelated individuals with qualified genotyping data from the China Kadoorie Biobank, the present study observed positive associations between snoring and total stroke, hemorrhagic stroke, and ischemic stroke. The associations remained stable in the MR among the non-obese group, and the multivariable MR analysis with adjusting for the body mass index. Our results indicated that the causal effect of snoring on stroke was independent of adiposity.

**Implications of all the available evidence**

Our findings suggest that compared with weight management, intervention in snoring through physical structure management, such as oropharyngeal exercises, and mandibular advancement devices, could be more beneficial to the prevention and control of stroke in the Chinese population.

## Introduction

With over two million new cases occurring every year, stroke is the leading cause of death among the Chinese,^1^ and the mortality has increased by 32.3% in the past 30 years.^2^ Snoring is a common problem among Chinese adults, with a prevalence of approximately 21.2% for habitual snoring.^3^ Compared to other sleep problems, snoring is more detectable. It could be more effectively interfered through weight loss,^4^ oropharyngeal exercises,^5^ mandibular advancement devices,^6^ etc.

A meta-analysis in 2015 included 126,427 participants from six cohort studies and reported a positive association between snoring and stroke.^7^ However, recent prospective studies didn't reach a consistent conclusion on the associations between snoring and different subtypes of stroke, such as hemorrhagic stroke (HS), and ischemic stroke (IS).^8^^,^^9^ Even though the conventional epidemiology studies were well-designed, prospective, and had a large-scale population, it was difficult to exclude stroke cases at an early stage completely through baseline questionnaires, which might lead to reverse associations.^9^ Unmeasured confounders (e.g., inflammatory markers^10^) might cause residual bias in the cohort studies. Considering the feasibility and ethical issues of snoring behavior intervention, it's impractical to conduct a randomized control trial (RCT).

Mendelian randomization (MR) is an alternative for causal inference. MR uses genetic variants as instrumental variables (IVs), which are robustly associated with the exposure, independent of confounders, and don't affect the outcomes via pathways other than the exposure. Thus, MR could efficiently reduce the reverse bias and residual confounding bias.^11^ However, no MR studies have been performed on snoring and stroke in East Asia so far. One of the key reasons is that the genetic variants for snoring among Asians are still unavailable. In addition, body mass index (BMI) was causal for the etiology of snoring according to the previous cross-lag^12^ and bidirectional MR analysis.^13^ Meanwhile, elevated BMI is a well-established factor for stroke.^14^ Therefore, the pleiotropy effect of BMI should be controlled for the causal estimation of snoring on stroke. Multivariable MR (MVMR) was designed to disentangle the closely correlated exposure and confounders, by incorporating IVs for both factors in the model.^15^

The present study conducted MR analysis to estimate the causal relationship between snoring and stroke as well as its subtypes among 82,339 participants from the China Kadoorie Biobank (CKB). Considering the potential pleiotropic effects caused by BMI, MR analysis among the non-obese participants (BMI<24.0 kg/m^2^) was conducted. In addition, snoring and BMI shared the genetic basis,^13^ MVMR adjusting for genetically predicted BMI was also applied to explore whether the effect of snoring was independent of BMI.

## Methods

### Study design and participants

The present study adhered to the STROBE-MR guidelines (Strengthening the Reporting of Observational Studies in Epidemiology using Mendelian Randomization) for reporting (STROBE-MR Checklist). The CKB cohort recruited 512,725 adults aged 30–79 years living in ten study areas across China. Extensive questionnaire data, physical measurements, and blood samples were collected upon baseline assessment in 2004–2008. Two batches of participants completed the genotyping procedures. Among them, 8143 had atherosclerotic vascular disease, 5917 had HS, 5203 had chronic obstructive pulmonary disease, and 81,377 were healthy controls. A total of 100,640 participants with both baseline and genotype data who passed the pre-imputation quality control (QC) were included.^16^^,^^17^ The CKB study was approved by the Ethics Review Committee of the Chinese Center for Disease Control and Prevention (Beijing, China) and the Oxford Tropical Research Ethics Committee, University of Oxford (Cambridge, UK). All participants provided written informed consent in the CKB study. A detailed description of the CKB study design could be found elsewhere.^16^^,^^17^

**Fig. 1:**
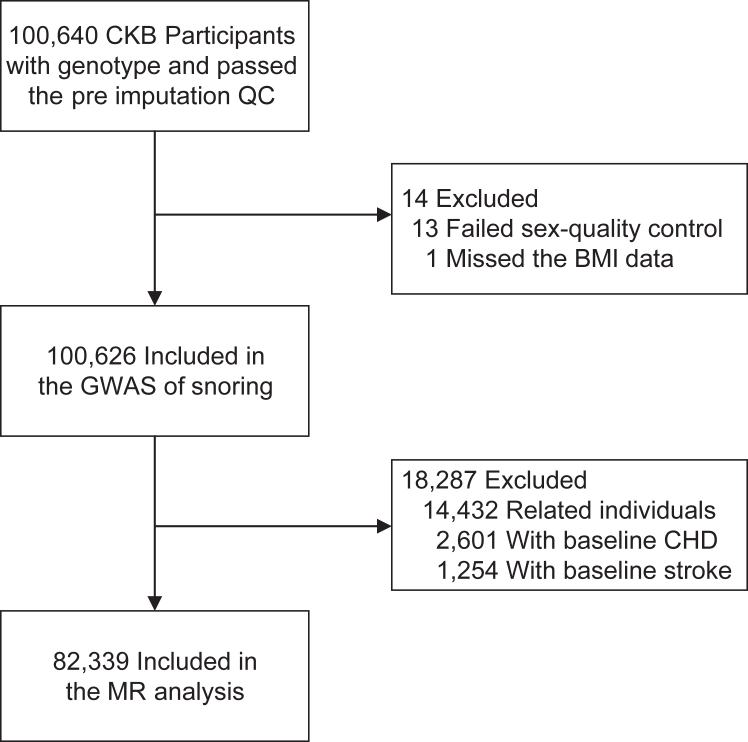
**Flow Diagram**. A flow diagram to show participants whose data were used in the genome-wide association study (GWAS) of snoring and Mendelian randomization (MR) analysis. CKB, China Kadoorie Biobank; QC, quality control; BMI, body mass index; CHD, coronary heart disease.

The present study excluded participants who failed the sex QC (n = 13) or with missing values (n = 1), leaving 100,626 participants for the genome-wide association study (GWAS) of snoring. We further excluded participants who reported a history of coronary heart disease (n = 2601), stroke (n = 1254) at baseline, and related individuals (n = 14,432, kinship coefficient >0.125), leaving 82,339 participants for MR analysis (Fig. 1).

### Snoring status and covariates

In the baseline survey, participants self-reported their snoring status as “Yes, frequently”, “Yes, sometimes”, or “No/Don't know.” Participants reported with the first two options were in the snoring group, and others were in the non-snoring group. Snoring showed good reproducibility among the 15,720 randomly selected participants to complete a repeated questionnaire survey within 1–2 weeks after the baseline (weighted-kappa = 0.69). The baseline questionnaire also collected demographic information (age, sex, study area, highest education), lifestyle factors (alcohol consumption, smoking status, total physical activity level-metabolic equivalent [MET] h/d), family history of stroke, diabetes, heart attack, and self-reported diagnoses of diabetes. Blood pressure, standing height and weight were obtained through physical measurement, and BMI was further calculated.

### Stroke

Incident cases of stroke were identified through linkages to disease and mortality registries as well as the national inpatient health insurance claim database, supplemented with local residential records and annual active confirmation. All events were coded according to the International Classification of Diseases, 10th version by trained staff blinded to baseline information. In the present study, three outcomes were stroke (I60–I69), HS (I61), and IS (I63). Since 2014, medical records of incident stroke cases were retrieved and reviewed by qualified cardiovascular specialists blinded to baseline exposures of patients. By October 2018, of 40,465 retrieved medical records of stroke cases, the diagnosis was confirmed in 91.8% of stroke cases,^18^ demonstrating the validity of the present registry and insurance-based definition.

### Genotyping, imputation, and quality control in CKB GWAS

Procedures for genotyping and imputation have been described previously.^19^, ^20^, ^21^ Genotyping was conducted using the Affymetrix Axiom array designed for Chinese Han ancestry. Qualified samples and genotypes were phased using SHAPEIT 3. The imputation was performed using IMPUTE 4 for each 5-Mb interval, taking the 1000 Genomes Phase III as the reference. The present study additionally performed the quality control of imputed single-nucleotide polymorphisms (SNPs). SNPs that met any of the following criteria^22^ were excluded: (i) information measure (Info) ≤0.3 for minor allele frequency (MAF) >3%, or Info ≤0.6 for MAF 1–3%, or Info ≤0.8 for MAF 0.5–1%, or Info ≤0.9 for MAF 0.1–0.5%; (2) P value for Hardy Weinberg equilibrium (P_HW_) ≤10^−6^; (3) call rate ≤95%; (4) SNPs on the sex chromosome; (5) SNPs on the major histocompatibility complex (MHC) regions. Besides, a detailed description of principal components (PC) analysis could be found elsewhere.^23^

### Construction of genetic risk score

A summary of the GRS constructed for the present study can be found in Supplementary Table S1. For UVMR analysis, genetic risk scores (GRSs) of snoring were constructed with SNPs selected from GWASs of CKB and UK Biobank (UKB), respectively (Supplementary Fig. S1).

An unweighted GRS was constructed based on the snoring GWAS in CKB (Supplementary Methods). Independent SNPs associated with snoring were included (P < 5 × 10^−8^, r^2^ < 0.001, window>10,000 kb, based on 1000 Genomes Project East sample [1000G EAS]), which also passed the replication from UKB GWAS (P < 5 × 10^−5^) (Supplementary Table S6). SNPs highly associated with BMI in CKB (P < 1 × 10^−5^) were excluded to limit the possible pleiotropic effect of BMI (Supplementary Table S9); two SNPs were left. GRS was calculated by summing the number of snoring probability-increasing alleles.

Considering that the winner's curse occurs if the IVs were initially discovered in the sample for MR analysis, and no other available snoring GWAS summary statistics in Asia, we constructed GRSs based on the snoring GWAS in UKB, an independent sample from CKB (Supplementary Methods). Independent snoring SNPs reported in the UKB GWAS were selected. SNPs that failed QC and were highly associated with BMI in CKB were excluded (Supplementary Tables S8 and S9), leaving 29 SNPs for the unweighted GRS. In addition, the weighted GRS of UKB was weighted by the reported effect sizes in UKB GWAS.^13^

Furthermore, to minimize the pleiotropy effect of BMI, all the SNPs associated with BMI in the CKB population (P < 0.05) were additionally excluded (Supplementary Table S9), leaving 18 SNPs identified in UKB GWAS for the unweighted GRS.

For the MVMR, GRSs of snoring and BMI were calculated. SNPs instrumented for snoring selected from CKB and UKB were applied. Besides, SNPs instrumented for BMI were selected from Biobank Japan (BBJ) (Supplementary Table S10).^24^ A total of 158,284 Japanese were included in the GWAS of BMI. Details of the GWAS analysis were shown in the Supplementary Methods.

PhenoScanner GWAS database^25^^,^^26^ was applied, and none of the SNPs (or proxy SNPs, r^2^ > 0.8) selected for the GRS construction were associated with stroke in previous studies.

### Two-stage MR analysis

The present study described baseline characteristics of participants in different CKB-unweighted GRS tertiles of snoring, which meant the low, intermediate, and high genetic risk of snoring, adjusting for sex, age, age^2^, study areas, the first ten PC1-10, genotyping array. The linear trend was tested by treating the GRS as a continuous variable.

The variance explained by the GRSs for snoring was estimated with the Efron pseudo-R^2^ value^27^ and *F* statistics.^28^ Efron pseudo-R^2^ of GRS (R^2^_GRS_) was the difference between full model R^2^ (logistic regression between snoring with GRS, sex, age, age^2^, study areas, PC1-10, and genotyping array) and null model R^2^ (full model without GRS). *F* statistic was equal to R^2^_GRS_ × sample size/(1- R^2^_GRS_). We tested the associations of potential confounders (highest education level [<9 years or ≥9 years], weekly drinking or not, current smoking or not, had a family history of stroke or not, BMI [<24.0 kg/m^2^ or ≥24.0 kg/m^2^], systolic blood pressure, diastolic blood pressure, total physical activity) with the GRS by the logistic or linear regression models.

A two-stage method was conducted to derive a population average causal hazard ratio. The first-stage model was a logistic regression of the GRS on snoring, and the second-stage model was a Cox regression of the genetically predicted probability of snoring on the outcome status. Both stages were adjusted for sex, age, age^2^, study areas, BMI, PC1-10, and genotyping array. Besides, The causal relationship among the non-obese participants (BMI<24.0 kg/m^2^) was examined.

In the MVMR analysis, the first-stage model was additionally adjusted for the GRS of BMI, and the second-stage model was additionally adjusted for the genetically predicted BMI. Both stages adjusted for the same covariates as above, except for BMI.^29^ The unweighted GRSs of CKB and UKB were applied for the main analysis, and the weighted GRS of UKB, the unweighted GRS of UKB without BMI SNPs were applied for the sensitivity analysis. Considering that snoring was a binary variable, the causal estimates were scaled to the average change in the risk of stroke per 0.5-fold increase in the genetically predicted probability of snoring (for example, an increase in the snoring probability from 20% to 30%) by multiplying the regression coefficient in the second stage by 0.405 (ln1.5).^30^

### MR with the inverse-variance weighted method

The inverse-variance weighted (IVW) method, which was suggested to safely examine the causal relationship in the presence of substantial confounding under the one-sample MR study design,^31^ was applied to estimate the association between snoring and stroke.^32^ Independent SNPs identified in the CKB GWAS and passed the replication were selected as the genetic IVs. The effect reported in the CKB GWAS was used as the effect of SNP on snoring. A logistic regression model was fitted to estimate the causal effect of SNP on the outcomes in the CKB population, adjusting for sex, age, age^2^, study areas, PC1-10, and genotyping array. The *F* statistic of each SNP was equal to the square of the genetic association with snoring divided by the square of its standard deviation [(*β*/SE)^2^], which was used to estimate the weak instrument bias (*F* < 10).^11^ The intercept of MR‒Egger regression^33^ was used to test the directional horizontal pleiotropy. Cochrane's Q test was performed to assess the heterogeneity among IVs, and a random-effect model in IVW was applied.^31^ In addition, a sensitivity analysis was conducted to exclude SNPs located on the *FTO* gene, which was known to be associated with adiposity.^34^

All of the statistical analyses were performed using Stata 16.0 and R software (version 4.1.0) with the “TwoSampleMR” package (version 0.5.6).^35^ The false discovery rate (FDR) was used to correct multiple tests.

### Role of the funding source

The funders had no role in the study design, data collection, data analysis and interpretation, writing of the report, or the decision to submit the article for publication. The corresponding author had full access to all the data in the study and took responsibility for its integrity and the data analysis.

### Ethics approval, consent to participate and consent to publish

The study protocol was approved by the Ethics Review Committee of the Chinese Center for Disease Control and Prevention (Beijing, China: 005/2004) and the Oxford Tropical Research Ethics Committee, University of Oxford (UK: 025–04). All participants provided written informed consent before taking part in the study.

## Results

### Associations between GRS and baseline characteristics

Among the 82,339 participants, 19,623 developed strokes, including 11,483 IS cases (58.5%) and 5710 HS cases (29.1%), during a median follow-up of 10.10 years (interquartile range 8.39–12.03 years).

As shown in Table 1, the highest CKB-snoring GRS group had the highest proportion of snoring at baseline (47.3%, P for trend <0.001), and the *F* statistics of the snoring GRS of CKB and UKB were between 25.46 and 59.10 (Supplementary Table S2). No significant differences were observed in other baseline characteristics across participants in the different GRS groups (FDR adjusted P for trend >0.05) (Table 1). Nor were the differences in the potential confounders (all P > 0.05) (Supplementary Table S4). In addition, *F* statistics of unweighted and weighted GRSs of BMI were 672.39 and 597.14, respectively (Supplementary Table S3). The GRSs of BMI were not significantly related to confounders (all P > 0.05) (Supplementary Table S4).

**Table 1:** Baseline characteristics of participants by CKB-GRS categories.

Characteristics	GRS of CKB			
	Low GRS	Intermediate GRS	High GRS	P for trend
N (%)	8065 (9.8)	23,831 (28.9)	50,443 (61.3)	
Snoring	44.5	45.7	47.3	<0.001
Male	41.7	41.3	41.7	0.954
Urban	43.7	44.3	44.3	0.578
Age (years)	53.6	53.7	53.5	0.115
≥9 y of education	47.3	47.2	47.6	0.698
Daily drinking	15.5	15.2	15.1	0.787
Current smoking	26.4	26.9	26.9	0.648
Total physical activity (MET h/d)	19.9	19.8	19.8	0.787
BMI (kg/m^2^)	23.6	23.6	23.6	0.698
SBP (mmHg)	133.0	133.2	133.4	0.414
DBP (mmHg)	78.5	78.5	78.5	0.578
Diabetes	7.2	6.5	6.4	0.115
Family history of diabetes	6.5	6.8	6.9	0.578
Family history of stroke	17.2	17.8	18.0	0.277
Family history of heart attack	3.5	3.2	3.3	0.992

**Notes:** BMI, body mass index; MET, metabolic equivalent of tasks; GRS, genetic risk scores; SBP, systolic blood pressure; DBP, diastolic blood pressure; CKB, China Kadoorie Biobank. Participants were divided into three groups according to their GRS of CKB. As for the P-value for trend, the GRS of CKB was treated as a continuous variable in the regression, adjusting for age, age^2^, sex, study areas, genetic array types, the first ten principal components, except for the age, sex, and study areas. The P-values were adjusted for false discovery rate.

### MR with a two-stage method

**Fig. 2:**
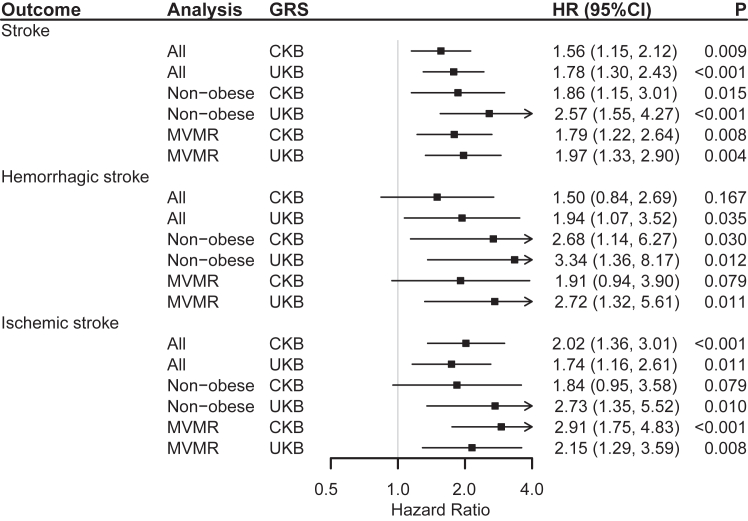
**Genetic Associations between Snoring and Stroke with two-stage Method**. **Notes:** GRS, genetic risk scores (all were unweighted); UKB, UK Biobank; CKB, China Kadoorie Biobank. MVMR, multivariable MR. Hazard ratios were scaled to represent the associations of a 0.5-fold increase in the prevalence of snoring and the average incident rate of outcomes. Both stages were adjusted for age, age^2^, sex, study areas, genetic array types, the first ten principal components, and body mass index (BMI). Non-obese group included participants with BMI <24.0 kg/m^2^. For MVMR analysis, the first stage was additionally adjusted for the GRS of BMI, the second stage was additionally adjusted for the genetically predicted BMI, and didn't adjust for BMI in both stages. The P-values were adjusted for false discovery rate.

Positive associations were between the genetically predicted probability of snoring and the outcomes, shown in Fig. 2. For the GRS of CKB, the HRs (95% CI) for genetically predicted per 0.5-fold increase in the probability of snoring with the risk of total stroke, HS, and IS were 1.56 (1.15, 2.12), 1.50 (0.84, 2.69), 2.02 (1.36, 3.01), respectively. For the GRS of UKB, the corresponding HRs were 1.78 (1.30, 2.43), 1.94 (1.07, 3.52), and 1.74 (1.16, 2.61).

Among the non-obese participants (BMI <24.0 kg/m^2^), GRS of CKB predicted probability of snoring remained significant association with total stroke (HR = 1.86, 95% CI: 1.15, 3.01) and HS (HR = 2.68, 95% CI: 1.14, 6.27). Besides, with adjusting for BMI in MVMR analysis, snoring was still positively associated with total stroke and IS, corresponding HRs (95% CIs) with GRS of CKB were 1.79 (1.22, 2.64) and 2.91 (1.75, 4.83), respectively (Fig. 2). In addition, sensitivity analysis with weighted GRS of UKB, and unweighted GRS of UKB without BMI-related SNPs, showed significant associations between the genetically predicted probability of snoring and total stroke and its subtypes (Supplementary Table S5).

### MR with the inverse-variance weighted method

**Fig. 3:**
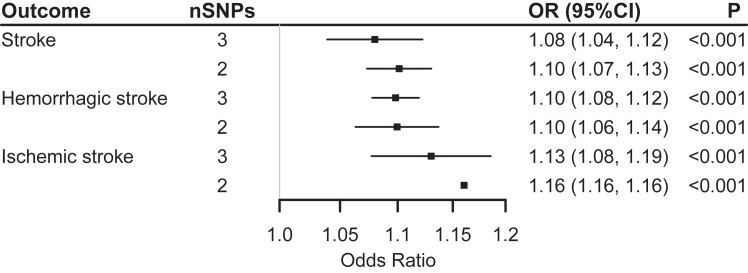
**Genetic Associations between Snoring and Stroke with Inverse-Variance Weighted (IVW) Method**. N**otes:** The three single-nucleotide polymorphisms (SNPs) of snoring were all from genome-wide association study of China Kadoorie Biobank. SNP rs8043757 on the *FTO* gene was excluded, leaving two SNPs for the sensitivity analysis. There was no significant pleiotropy in the test for the intercept of MR-Egger regression (P > 0.05). The P-values were adjusted for false discovery rate.

IVW method also showed positive causal estimates of snoring on stroke. Three SNPs identified in the CKB GWAS were used as the genetic IVs for snoring (Supplementary Table S6). The genetically predicted 0.5-fold increase in the probability of snoring was associated with increased risk of stroke, HS, and IS. The corresponding ORs were 1.08 (1.04, 1.12), 1.10 (1.08, 1.12), and 1.13 (1.08, 1.19), respectively (Fig. 3).

Limited evidence indicated the weak instrument bias, all *F*-statistics for the three SNPs were more than ten. The test of the MR‒Egger regression intercept showed no significant horizontal pleiotropy (P > 0.05). The direction of snoring to outcomes was significant (MR-Steiger test P < 0.001), and there was no heterogeneity (Cochrane's Q test P > 0.05) (Supplementary Table S7). In addition, the causal estimates between snoring and the outcomes remained significant when the SNP located on the *FTO* gene was further excluded (all P < 0.001) (Fig. 3).

## Discussion

Based on the MR analyses of a large sample of individual-level data from CKB, snoring was consistently shown as a potential causal factor for the increased risk of total stroke, HS, and IS among Chinese adults. Furthermore, such causal associations remained stable, independent of the effect of BMI.

Previous cohort studies have estimated the association between snoring and stroke. Titova et al. found that disturbing snoring was associated with intracerebral hemorrhage (1.59 [1.23–2.05]) but not with IS (1.06 [0.95–1.19]).^8^ In contrast, our previous work based on 0.5 million CKB participants found that habitual snoring was associated with increased risks of IS (1.12 [1.05–1.19]), but not HS (1.05 [0.92–1.19]), while the association was not significant among the non-obese participants.^9^ Reverse bias might account for the differences between traditional and the present MR studies. Considering the high prevalence of sleep-disorder breathing problems after stroke,^36^ previous cohort studies probably included participants at the early stage of stroke, even though participants with clinically diagnosed stroke were excluded. Notably, residual or inaccurately measured confounders, such as inflammatory markers^10^ could also mask the association between snoring and stroke in the observational studies.

The present study was the first MR study evaluating the causal relationship between snoring and stroke among the Asian population, partly due to a lack of available genetic variants. Two previous two-sample MR studies were performed among the Europeans, while the results were inconsistent, probably influenced by the difference in the sample sizes of the discovery population of snoring GWAS.^37^^,^^38^ The latest study, performed based on a recent meta-analysis of the GWAS of snoring (172,050 snoring cases and 350,316 controls), whose sample size was larger than another study, didn't observe an association between snoring and stroke, both in UVMR (OR [95% CI] = 1.06 [0.84–1.34]) and MVMR with adjusting for BMI (1.03 [0.77–1.37]).^37^ The ethnic difference might contribute to the difference between the present and previous studies. Based on GWAS of snoring among 100,626 CKB participants, our study selected independent SNPs associated with snoring at a genome-wide significant level as genetic instruments, which could properly evaluate the causal effect of snoring on stroke among the Chinese. In addition, the proportions of stroke cases were different between the Western and Chinese populations. The previous studies used the MEGASTROKE consortium, with 9.1% and 8.4% of participants developing stroke or IS,^39^ the proportions were far less than that of CKB (total stroke: 23.8%, IS: 13.9%), which could lead to the difference in causal estimation.

Adiposity was a potential confounder in the relationship between snoring and stroke. Our previous work reported that baseline BMI could increase habitual snoring at resurvey.^12^ MR analysis based on UKB participants also supported a causal effect of higher BMI on the risk of snoring.^13^ When the fat is deposited in surrounding structures of the upper airway or within the tongue, it could lead to airway collapse and cause snoring.^40^ The present study conducted a series of analyses to address the confounding bias from adiposity, i.e., constructing the GRS independent from BMI, re-running the MR analysis in the non-obese group, and using the MVMR model to adjust BMI. The robust results indicated that the causal effect of snoring on stroke was independent of BMI pleiotropy. Thus, compared to weight management, intervention in snoring through physical structure management was probably more beneficial to preventing stroke. Previous RCTs showed that oropharyngeal exercises^5^ and mandibular advancement devices^6^ were effective for snoring treatment.

The mechanisms underlying the causal linkage between snoring and stroke may involve several pathways. Snoring could cause anoxemia, leading to oxidative stress responses and endothelial dysfunction,^41^ all of which might lead to atherosclerosis and contribute to incident stroke.^42^ In addition, a high-level vibration transmitted to the carotid artery caused by snoring might trigger a cascade effect on the numerous cells of the arterial wall and lead to injury to the vessel and rupture of plaque.^42^

To our knowledge, we were the first study to investigate the causal relationship between snoring and total stroke, HS, and IS among the Asian population. The present study used genetic risk loci for snoring identified in Chinese participants and replicated in the external population, which were more suitable IVs for MR analysis in China. Furthermore, MR analysis was conducted based on a negative control design in the non-obese participants, as well as MVMR adjusting for genetically predicted BMI to reduce the pleiotropy of BMI.

However, there are several limitations to mention. First, the genetically instrumented snoring in the present study might not comprehensively characterize the etiology of snoring and the effect of snoring on stroke. Meanwhile, the *F* statistics were more than ten, suggesting a small magnitude of the weak instrument bias. Additionally, the causal estimates of snoring on stroke were broadly consistent across different MR analyses, indicating that the selection of IVs was appropriate. And the unweighted GRS of CKB was applied to minimize the potential weak instrument bias.^11^^,^^43^ Second, considering using the GRS of CKB might lead to the winner's curse, loci reported in the independent UKB population were additionally applied, and the weights derived from UKB were used.^11^ Third, snoring status was self-reported and might suffer from information bias. However, the misclassification tended to be non-differential, leading to the results toward the null hypotheses.^44^ Future studies could perform objective measurements (e.g., polysomnography) for snoring in CKB. Fourth, unmeasured factors, such as chronic inflammation and metabolic markers, limited further investigation of the potential mechanisms between snoring and stroke. Besides, due to the different types of stroke outcomes between CKB and available summary statistics from Biobank of Japan,^45^ we didn't replicate MR analysis with a two-sample MR design in the Asian population. Finally, the CKB study was not designed to represent China's general population. Therefore, caution must be taken in generalizing our findings to the broader Chinese population.

### Conclusion

In summary, the present study suggested causal relationships that snoring could increase the risks of total stroke, IS, and HS. The causal effects were independent of BMI. Our findings indicated that compared with BMI management, intervention in snoring through the physical structure treatment, such as mandibular advancement devices, could be more beneficial to preventing and controlling stroke in the Chinese population.

## Contributors

LL, JL conceived and designed the study. LL, ZC, and JC, members of the China Kadoorie Biobank Steering Committee, designed and supervised the whole study, obtained funding, and, together with CY, DSun, PP, LY, YC, HD, IM, RW, XW, DSchmidt, DA acquired the data. YZ and ZZ analyzed the data. YZ drafted the manuscript. CY helped to interpret the results. CY contributed to the critical revision of the manuscript for important intellectual content. All authors reviewed and approved the final manuscript. CY had full access to all the data in the study and took responsibility for its integrity and the data analysis.

## Data sharing statement

The access policy and procedures are available at www.ckbiobank.org.

## Declaration of interests

The authors have no relevant financial or non-financial interests to disclose.

## Appendix A Supplementary data

Supplementary Figure S2

Translated Abstract
