# Supplementary material for: Causal association between snoring and stroke: a Mendelian randomization study in a Chinese population
Source: Lancet Reg Health West Pac. 2024 Jan 23;44:101001. doi: 10.1016/j.lanwpc.2023.101001 (PMC10832459; doi:10.1016/j.lanwpc.2023.101001)
Supplement: Supplementary Material [file mmc1.docx]

**Causal Association between Snoring and Stroke: A Mendelian Randomization Study in a Chinese Population**

Yunqing Zhu, MSc^a^, Zhenhuang Zhuang, BSc^a^, Jun Lv, PhD^a,b,c^, Dianjianyi Sun, PhD^a,b,c^, Pei Pei, BSc^b^, Ling Yang, PhD^d,e^, Iona Y. Millwood, DPhil^d,e^, Robin G.Walters, PhD^d,e^, Yiping Chen, DPhil^d,e^, Huaidong Du, PhD^d,e^, Xianping Wu, MSc^f^, Dan Schmidt, MSc^e^, Daniel Avery, MSc^e^, Junshi Chen, MD^g^, Zhengming Chen, DPhil^e^, Liming Li, MPH^a,b,c^, Canqing Yu, PhD^a,b,c*^, on behalf of the China Kadoorie Biobank Collaborative Group^†^

^a^ Department of Epidemiology & Biostatistics, School of Public Health, Peking University, Beijing 100191, China;

^b^ Peking University Center for Public Health and Epidemic Preparedness & Response, Beijing 100191, China;

^c^ Key Laboratory of Epidemiology of Major Diseases (Peking University), Ministry of Education, Beijing 100191, China;

^d^ Medical Research Council Population Health Research Unit at the University of Oxford, Oxford OX3 7LF, United Kingdom;

^e^ Clinical Trial Service Unit & Epidemiological Studies Unit (CTSU), Nuffield Department of Population Health, University of Oxford OX3 7LF, United Kingdom;

^f^ Suzhou Centers for Disease Control, NO.72 Sanxiang Road, Gusu District, Suzhou 215004, Jiangsu, China;

^g^ China National Center for Food Safety Risk Assessment, Beijing 100022, China

**^*^Corresponding author:**

Canqing Yu, PhD

Department of Epidemiology and Biostatistics

Peking University Health Science Center

38 Xueyuan Road, Beijing 100191, China

Phone: 86-10-82801528

Email: yucanqing@pku.edu.cn

^†^The members of steering committee and collaborative group are listed in the Supplementary Material.

**Supplementary Methods**

**GWAS of snoring in CKB**

The genome-wide association study (GWAS) analysis for snoring in China Kadoorie Biobank (CKB, n = 100,626, 47,208 snoring cases) was conducted using BOLT-LMM 2.3.2 linear mixed model[1]. 7,063,876 genotyped or imputed single-nucleotide polymorphisms (SNPs) were analyzed in additive genetic models adjusting for age, age^2^, sex, study areas, genotyping array, the first ten principal components (PC1-10) of ancestry at the national level, and four baseline disease statuses[2]. And we conducted a transformation to convert SNP effect size estimates (*β*) on the quantitative scale to traditional *β* when analyzing case-control traits[3].

To test the validity of our results, an independent replication was performed in the UK Biobank (UKB) GWAS of snoring, with a sample size of 408,317 participants (152,000 snoring cases) [4]. We confirmed that a genomic risk locus passed the replication if the locus (i) existed in UKB GWAS summary statistics; (ii) the direction of *β* and effect allele was matched across CKB and UKB. (iii) with a P-value < 5.00×10^-5^ in UKB GWAS.

**GWAS of snoring in UKB**

The UKB study design, procedures of genotyping, imputation, and quality control (QC) before analysis were described in detail elsewhere[5]. The habitual snoring GWAS study included participants of European ancestry. Baseline data were collected between 2006-2010. Snoring was a self-reported trait (Field-ID: 1210): “Does your partner or a close relative or friend complain about your snoring?” After excluding participants who answered “Don’t know” or “Prefer not to answer”, a total of 408,000 participants were included in the GWAS, containing 37% snorers. GWAS of snoring in UKB was performed using BOLT-LMM, adjusting for age, sex, genotyping array, and the first 20 PCs as a fixed effect. A post-GWAS strict QC was carried out, corresponding to minor allele frequency ≥0.005 and imputation quality score ≥0.6[4].

**BBJ BMI GWAS dataset**

The Biobank Japan (BBJ) study design, procedures of genotyping, imputation, and QC before analysis were described in detail elsewhere[6, 7]. Briefly, a total of 158,284 participants were included in the body mass index (BMI) GWAS study in BBJ, after sample QC of age ≥18, weight and height data registered and within threefold the interquartile range, call rate >0.98, not closely related sample, not outliers in PC analysis. BMI in the BBJ study was obtained from medical records, the log-transformed BMI was adjusted for age, age^2^, sex, 47 registered diseases, and PC1-10. The resulting residual was transformed using a rank-based inverse-normal transformation. BMI GWAS data were imputed with the East Asian sample of 1000 Genomes Project Phase I v3 reference panel. QC in BBJ used the following criteria: sample call rate <0.98, SNP call rate <0.99, P value for Hardy Weinberg equilibrium (P_HW_) ≤10^-6^, and imputation quality score <0.7 were excluded [2].

**GRS for MVMR**

For the multivariable Mendelian randomization (MVMR) analysis, we calculated the genetic risk scores (GRS) for snoring and GRS for BMI. Compared with the GRS in univariable MR, snoring GRS (CKB-unweighted, UKB-weighted, and UKB-unweighted) for MVMR didn’t exclude the SNPs highly associated with BMI, 3 and 11 SNPs were respectively used for GRS of CKB and UKB.

As for GRS of BMI, the independent SNPs from the BMI GWAS of BBJ (P < 5 × 10^-8^, r^2^ < 0.001, window < 10000 kb in East Asia)[8] were selected. And we excluded SNPs that failed QC, failed replication, or SNPs highly associated with potential confounders in CKB (P < 1 × 10^-5^). 44 SNPs were left (Supplementary Fig S2, Supplementary Table S10). Then the unweighted and weighted GRSs of BMI were calculated, the latter was weighted by the effect reported in BBJ GWAS[8].

R^2^ explained by BMI GRS (R^2^_GRS_) and *F* statistics were calculated to estimate the performance of GRS on BMI. R^2^_GRS_ was equal to the differences between R^2^ in the full model (linear regression of BMI GRS on BMI, adjusting for sex, age, age^2^, study areas, PC1-10, and genotyping array) with R^2^ of the null model (full model without GRS). *F* statistics was equal to R^2^_GRS_×sample size / (1- R^2^_GRS_)[9].

**Members of the China Kadoorie Biobank collaborative group**

**International Steering Committee:** Junshi Chen, Zhengming Chen (PI), Robert Clarke, Rory Collins, Yu Guo, Liming Li (PI), Jun Lv, Richard Peto, Robin Walters. **International Co-ordinating Centre, Oxford:** Daniel Avery, Derrick Bennett, Ruth Boxall, Sue Burgess, Ka Hung Chan, Yumei Chang, Yiping Chen, Zhengming Chen, Johnathan Clarke; Robert Clarke, Huaidong Du, Ahmed Edris Mohamed, Zammy Fairhurst-Hunter, Hannah Fry, Mike Hill, Michael Holmes, Pek Kei Im, Andri Iona, Maria Kakkoura, Christiana Kartsonaki, Rene Kerosi, Kuang Lin, Mohsen Mazidi, Iona Millwood, Sam Morris, Qunhua Nie, Alfred Pozarickij, Paul Ryder, Saredo Said, Dan Schmidt, Paul Sherliker, Becky Stevens, Iain Turnbull, Robin Walters, Lin Wang, Neil Wright, Ling Yang, Xiaoming Yang, Pang Yao.

**National Co-ordinating Centre, Beijing:** Yu Guo, Xiao Han, Can Hou, Jun Lv, Pei Pei, Chao Liu, Canqing Yu, Qingmei Xia. **10 Regional Co-ordinating Centres: Qingdao CDC:** Zengchang Pang, Ruqin Gao, Shanpeng Li, Haiping Duan, Shaojie Wang, Yongmei Liu, Ranran Du, Yajing Zang, Liang Cheng, Xiaocao Tian, Hua Zhang, Yaoming Zhai, Feng Ning, Xiaohui Sun, Feifei Li. **Licang CDC:** Silu Lv, Junzheng Wang, Wei Hou. **Heilongjiang Provincial CDC:** Wei Sun, Shichun Yan, Xiaoming Cui. **Nangang CDC:** Chi Wang, Zhenyuan Wu,Yanjie Li, Quan Kang. **Hainan Provincial CDC:** Huiming Luo, Tingting Ou. **Meilan CDC:** Xiangyang Zheng, Zhendong Guo, Shukuan Wu, Yilei Li, Huimei Li. **Jiangsu Provincial CDC:** Ming Wu, Yonglin Zhou, Jinyi Zhou, Ran Tao, Jie Yang, Jian Su. **Suzhou CDC:** Fang Liu, Jun Zhang, Yihe Hu, Yan Lu, Liangcai Ma, Aiyu Tang, Shuo Zhang, Jianrong Jin, Jingchao Liu. **Guangxi Provincial CDC:** Mei Lin, Zhenzhen Lu. **Liuzhou CDC:** Lifang Zhou, Changping Xie, Jian Lan,Tingping Zhu,Yun Liu, Liuping Wei, Liyuan Zhou, Ningyu Chen, Yulu Qin, Sisi Wang. **Sichuan Provincial CDC:** Xianping Wu, Ningmei Zhang, Xiaofang Chen, Xiaoyu Chang. **Pengzhou CDC:** Mingqiang Yuan, Xia Wu, Xiaofang Chen, Wei Jiang, Jiaqiu Liu, Qiang Sun. **Gansu Provincial CDC:** Faqing Chen, Xiaolan Ren, Caixia Dong. **Maiji CDC:** Hui Zhang, Enke Mao, Xiaoping Wang, Tao Wang, Xi zhang. **Henan Provincial CDC:** Kai Kang, Shixian Feng, Huizi Tian, Lei Fan. **Huixian CDC:** XiaoLin Li, Huarong Sun, Pan He, Xukui Zhang. **Zhejiang Provincial CDC:** Min Yu, Ruying Hu, Hao Wang. **Tongxiang CDC**: Xiaoyi Zhang, Yuan Cao, Kaixu Xie, Lingli Chen, Dun Shen. **Hunan Provincial CDC:** Xiaojun Li, Donghui Jin, Li Yin, Huilin Liu, Zhongxi Fu. **Liuyang CDC:** Xin Xu, Hao Zhang, Jianwei Chen,Yuan Peng, Libo Zhang, Chan Qu.

**References**

1. Loh PR, Tucker G, Bulik-Sullivan BK, Vilhjálmsson BJ, Finucane HK, Salem RM et al. Efficient Bayesian mixed-model analysis increases association power in large cohorts. Nat Genet. 2015;47(3):284-90.doi:10.1038/ng.3190

2. Akiyama M, Okada Y, Kanai M, Takahashi A, Momozawa Y, Ikeda M et al. Genome-wide association study identifies 112 new loci for body mass index in the Japanese population. Nat Genet. 2017;49(10):1458-67.doi:10.1038/ng.3951

3. Loh P-R. BOLT-LMM v2.3.5 User Manual 2021 [Available from: <https://storage.googleapis.com/broad-alkesgroup-public/BOLT-LMM/BOLT-LMM_manual.html#x1-5300010>.

4. Campos AI, García-Marín LM, Byrne EM, Martin NG, Cuéllar-Partida G, Rentería ME. Insights into the aetiology of snoring from observational and genetic investigations in the UK Biobank. Nat Commun. 2020;11(1):817.doi:10.1038/s41467-020-14625-1

5. Bycroft C, Freeman C, Petkova D, Band G, Elliott LT, Sharp K et al. The UK Biobank resource with deep phenotyping and genomic data. Nature. 2018;562(7726):203-9.doi:10.1038/s41586-018-0579-z

6. Nagai A, Hirata M, Kamatani Y, Muto K, Matsuda K, Kiyohara Y et al. Overview of the BioBank Japan Project: Study design and profile. J Epidemiol. 2017;27(3s):S2-s8.doi:10.1016/j.je.2016.12.005

7. Nakamura YJCAHO. The BioBank Japan Project. 2007;5:696-7

8. Ishigaki K, Akiyama M, Kanai M, Takahashi A, Kawakami E, Sugishita H et al. Large-scale genome-wide association study in a Japanese population identifies novel susceptibility loci across different diseases. Nat Genet. 2020;52(7):669-79.doi:10.1038/s41588-020-0640-3

9. Pang Y, Kartsonaki C, Lv J, Fairhurst-Hunter Z, Millwood IY, Yu C et al. Associations of Adiposity, Circulating Protein Biomarkers, and Risk of Major Vascular Diseases. JAMA Cardiol. 2021;6(3):276-86.doi:10.1001/jamacardio.2020.6041
