# Supplementary material for: Causal association between snoring and stroke: a Mendelian randomization study in a Chinese population
Source: Lancet Reg Health West Pac. 2024 Jan 23;44:101001. doi: 10.1016/j.lanwpc.2023.101001 (PMC10832459; doi:10.1016/j.lanwpc.2023.101001)
Supplement: Supplementary Figure Legends and Tables [file mmc2.docx]

**Causal Association between Snoring and Stroke: A Mendelian Randomization Study in a Chinese Population**

Yunqing Zhu, MSc^a^, Zhenhuang Zhuang, BSc^a^, Jun Lv, PhD^a,b,c^, Dianjianyi Sun, PhD^a,b,c^, Pei Pei, BSc^b^, Ling Yang, PhD^d,e^, Iona Y. Millwood, DPhil^d,e^, Robin G.Walters, PhD^d,e^, Yiping Chen, DPhil^d,e^, Huaidong Du, PhD^d,e^, Xianping Wu, MSc^f^, Dan Schmidt, MSc^e^, Daniel Avery, MSc^e^, Junshi Chen, MD^g^, Zhengming Chen, DPhil^e^, Liming Li, MPH^a,b,c^, Canqing Yu, PhD^a,b,c*^, on behalf of the China Kadoorie Biobank Collaborative Group^†^

^a^ Department of Epidemiology & Biostatistics, School of Public Health, Peking University, Beijing 100191, China;

^b^ Peking University Center for Public Health and Epidemic Preparedness & Response, Beijing 100191, China;

^c^ Key Laboratory of Epidemiology of Major Diseases (Peking University), Ministry of Education, Beijing 100191, China;

^d^ Medical Research Council Population Health Research Unit at the University of Oxford, Oxford OX3 7LF, United Kingdom;

^e^ Clinical Trial Service Unit & Epidemiological Studies Unit (CTSU), Nuffield Department of Population Health, University of Oxford OX3 7LF, United Kingdom;

^f^ Suzhou Centers for Disease Control, NO.72 Sanxiang Road, Gusu District, Suzhou 215004, Jiangsu, China;

^g^ China National Center for Food Safety Risk Assessment, Beijing 100022, China

**^*^Corresponding author:**

Canqing Yu, PhD

Department of Epidemiology and Biostatistics

Peking University Health Science Center

38 Xueyuan Road, Beijing 100191, China

Phone: 86-10-82801528

Email: yucanqing@pku.edu.cn

^†^The members of steering committee and collaborative group are listed in the Supplementary Material.

**Supplementary Figure Legends and Tables.**

[Supplementary Figure Legends 4](#_Toc155985111)

[Supplementary Fig S1 Selection of Snoring SNPs and Construction of GRS for Snoring in CKB 4](#_Toc155985112)

[Supplementary Fig S2 Selection of BMI SNPs and Construction of GRS for BMI in CKB 4](#_Toc155985113)

[Supplementary Tables 5](#_Toc155985114)

[Supplementary Table S1. A summary of the genetic risk scores (GRS) calculated in the present MR study. 5](#_Toc155985115)

[Supplementary Table S2. Associations between snoring GRS and snoring in CKB. 6](#_Toc155985116)

[Supplementary Table S3. Associations between BMI GRS and BMI in CKB. 7](#_Toc155985117)

[Supplementary Table S4. Associations between GRS and Potential Confounders. 8](#_Toc155985118)

[Supplementary Table S5. Sensitivity analysis using two-stage method. 10](#_Toc155985119)

[Supplementary Table S6. SNPs for snoring identified in CKB GWAS and the replication in UKB GWAS. 11](#_Toc155985120)

[Supplementary Table S7. Tests for the MR analysis. 12](#_Toc155985121)

[Supplementary Table S8. SNPs for snoring identified in UKB GWAS. 13](#_Toc155985122)

[Supplementary Table S9. Genetic associations between snoring SNPs and BMI in CKB. 15](#_Toc155985123)

[Supplementary Table S10. SNPs for body mass index (BMI) identified in BBJ GWAS. 17](#_Toc155985124)

**Supplementary Figure Legends**

**Supplementary Fig S1 Selection of Snoring SNPs and Construction of GRS for Snoring in CKB**

**Notes:** GRS, genetic risk scores; UKB, UK Biobank; CKB, China Kadoorie Biobank; BMI, body mass index; QC, quality control; SNP, single-nucleotide polymorphisms. Criteria for CKB GWAS QC included: (i) Information measure (Info) ≤ 0.3 for minor allele frequency (MAF) > 3%, or Info ≤ 0.6 for MAF 1-3%, or Info ≤ 0.8 for MAF 0.5-1%, or Info ≤ 0.9 for MAF 0.1-0.5%; (2) P value for Hardy Weinberg equilibrium test ≤ 10^-6^; (3) call rate ≤ 95%; (4) SNPs on the sex chromosome; (5) SNPs on the major histocompatibility complex regions (chr6: 25-34M).

**Supplementary Fig S2 Selection of BMI SNPs and Construction of GRS for BMI in CKB**

**Notes:** GRS, genetic risk scores; BBJ, Biobank Japan; CKB, China Kadoorie Biobank; BMI, body mass index; QC, quality control; SNP, single-nucleotide polymorphisms; LD, linkage disequilibrium. Criteria for CKB GWAS QC included: (i) Information measure (Info) ≤ 0.3 for minor allele frequency (MAF) > 3%, or Info ≤ 0.6 for MAF 1-3%, or Info ≤ 0.8 for MAF 0.5-1%, or Info ≤ 0.9 for MAF 0.1-0.5%; (2) P value for Hardy Weinberg equilibrium test ≤ 10^-6^; (3) call rate ≤ 95%; (4) SNPs on the sex chromosome; (5) SNPs on the major histocompatibility complex regions (chr6: 25-34M).

**Supplementary Tables**

**Supplementary Table S1. A summary of the genetic risk scores (GRS) calculated in the present MR study.**

| **UVMR** | **Unweighted GRS** | **Weighted GRS-weighted by** | **Excluded BMI SNPs** |
| --- | --- | --- | --- |
| CKB-Snoring | 2 | Winner's curse | No SNPs left |
| UKB-Snoring | 29 | 29-UKB snoring GWAS | 18 |
| **MVMR** | **Unweighted GRS** | **Weighted GRS-weighted by** | **Excluded BMI SNPs** |
| CKB-Snoring | 2 | Winner's curse | BMI was conditioned in MVMR analysis |
| UKB-Snoring | 30 | 30-UKB snoring GWAS |  |
| BBJ-BMI | 44 | 44-BBJ BMI GWAS |  |

**Notes:** GRS, genetic risk scores; UVMR, univariable MR; MVMR, multivariable MR; CKB, China Kadoorie Biobank; UKB, UK Biobank; BBJ, Biobank of Japan; BMI, body mass index; GWAS, genome-wide association study.

The based samples for GWAS and the corresponding traits were shown in the first column. The numbers of SNPs used for GRS construction were shown in the table, and the base GWASs of the weights were shown in the corresponding cells of weighted GRSs.

**Supplementary Table S2. Associations between snoring GRS and snoring in CKB.**

| **GRS** | **Unweighted GRS of CKB** | **Unweighted GRS of UKB** | **Weighted GRS of UKB** | **Unweighted GRS of UKB without BMI SNPs** |
| --- | --- | --- | --- | --- |
| **UVMR** |  |  |  |  |
| Number of SNPs | 2 | 29 | 29 | 18 |
| Odds ratio (95%CI) | 1.06 (1.04 ,1.07) | 1.02 (1.01 ,1.02) | 1.02 (1.01 ,1.02) | 1.02 (1.01 ,1.02) |
| *F* statistics | 49.05 | 46.55 | 52.52 | 25.46 |
| Efron Pseudo R^2^_GRS_ | 0.06% | 0.06% | 0.06% | 0.03% |
| **MVMR** |  |  |  |  |
| Number of SNPs | 2 | 30 | 30 | NA |
| Odds ratio (95%CI) | 1.06 (1.04 ,1.07) | 1.02 (1.01 ,1.02) | 1.02 (1.01 ,1.02) | NA |
| *F* statistics | 49.05 | 53.79 | 59.10 | NA |
| Efron Pseudo R^2^_GRS_ | 0.06% | 0.07% | 0.07% | NA |

**Notes:** GRS, genetic risk scores; UKB, UK Biobank; CKB, China Kadoorie Biobank; SNP, single-nucleotide polymorphisms; BMI, body mass index.

Efron pseudo-R^2^ of GRS (R^2^_GRS_) was the difference between full model R^2^ (logistic regression between snoring with GRS, sex, age, age^2^, study areas, the first ten principal components , and genotyping array) and null model R^2^ (full model without GRS). *F* statistics was equal to R^2^_GRS_×sample size / (1- R^2^_GRS_).

**Supplementary Table S3. Associations between BMI GRS and BMI in CKB.**

|  | **Unweighted BMI GRS** | **Weighted BMI GRS** |
| --- | --- | --- |
| Number of SNPs | 44 | 44 |
| *β* (95%CI) | 0.071 (0.066, 0.077) | 0.079 (0.073, 0.084) |
| *F* Statistics | 672.39 | 597.14 |
| R^2^_GRS_ | 0.81% | 0.72% |

**Notes:** GRS, genetic risk scores; CKB, China Kadoorie Biobank; BMI, body mass index; R^2^_GRS_ was equal to the differences between R^2^ in full model (linear regression between BMI and the GRS of BMI, adjusting for sex, age, age^2^, study areas, the first ten principal components, and genotyping array) with R^2^ of null model (full model without GRS). *F* statistic was equal to R^2^_GRS_×sample size / (1- R^2^_GRS_).

**Supplementary Table S4. Associations between GRS and Potential Confounders.**

| **GRS** | **Confounders** | **Coef (95%CI)** | **P** |
| --- | --- | --- | --- |
| **GRS for UVMR** |  |  |  |
| CKB-unweighted |  |  |  |
|  | Education | 1.01 (0.99, 1.03) | 0.797 |
|  | Weekly drinking | 0.99 (0.97, 1.01) | 0.797 |
|  | Current smoking | 1.01 (0.99, 1.03) | 0.797 |
|  | Family history of stroke | 1.02 (1.00, 1.04) | 0.489 |
|  | BMI | 1.00 (0.99, 1.02) | 0.948 |
|  | Systolic blood pressure | 0.118 (-0.038, 0.274) | 0.660 |
|  | Diastolic blood pressure | 0.044 (-0.041, 0.129) | 0.797 |
|  | Total physical activity | -0.018 (-0.104, 0.068) | 0.877 |
| UKB-weighted |  |  |  |
|  | Education | 1.00 (0.99, 1.00) | 0.879 |
|  | Weekly drinking | 1.00 (0.99, 1.01) | 0.877 |
|  | Current smoking | 1.00 (1.00, 1.01) | 0.797 |
|  | Family history of stroke | 1.00 (0.99, 1.00) | 0.797 |
|  | BMI | 1.00 (1.00, 1.01) | 0.849 |
|  | Systolic blood pressure | 0.048 (-0.000, 0.097) | 0.488 |
|  | Diastolic blood pressure | 0.011 (-0.015, 0.037) | 0.797 |
|  | Total physical activity | -0.002 (-0.028, 0.025) | 0.958 |
| UKB-unweighted |  |  |  |
|  | Education | 1.00 (0.99, 1.01) | 0.948 |
|  | Weekly drinking | 1.00 (0.99, 1.01) | 0.948 |
|  | Current smoking | 1.00 (1.00, 1.01) | 0.797 |
|  | Family history of stroke | 1.00 (0.99, 1.00) | 0.797 |
|  | BMI | 1.00 (1.00, 1.01) | 0.849 |
|  | Systolic blood pressure | 0.057 (0.009, 0.104) | 0.246 |
|  | Diastolic blood pressure | 0.018 (-0.007, 0.044) | 0.719 |
|  | Total physical activity | -0.001 (-0.027, 0.025) | 0.961 |
| UKB-withoutBMI-uw |  |  |  |
|  | Education | 1.00 (1.00, 1.01) | 0.797 |
|  | Weekly drinking | 1.00 (0.99, 1.01) | 0.993 |
|  | Current smoking | 1.00 (0.99, 1.01) | 0.849 |
|  | Family history of stroke | 1.00 (0.99, 1.00) | 0.797 |
|  | BMI | 1.00 (0.99, 1.01) | 0.958 |
|  | Systolic blood pressure | 0.078 (0.018, 0.138) | 0.201 |
|  | Diastolic blood pressure | 0.042 (0.010, 0.075) | 0.201 |
|  | Total physical activity | -0.016 (-0.049, 0.017) | 0.797 |
| **GRS for MVMR** |  |  |  |
| CKB-unweighted |  |  |  |
|  | Education | 1.01 (0.99, 1.03) | 0.797 |
|  | Weekly drinking | 0.99 (0.97, 1.01) | 0.797 |
|  | Current smoking | 1.01 (0.99, 1.03) | 0.797 |
|  | Family history of stroke | 1.02 (1.00, 1.04) | 0.489 |
|  | Systolic blood pressure | 0.118 (-0.038, 0.274) | 0.660 |
|  | Diastolic blood pressure | 0.044 (-0.041, 0.129) | 0.797 |
|  | Total physical activity | -0.018 (-0.104, 0.068) | 0.877 |
| UKB-unweighted |  |  |  |
|  | Education | 1.00 (0.99, 1.00) | 0.849 |
|  | Weekly drinking | 1.00 (0.99, 1.01) | 0.993 |
|  | Current smoking | 1.00 (1.00, 1.01) | 0.797 |
|  | Family history of stroke | 1.00 (0.99, 1.01) | 0.948 |
|  | Systolic blood pressure | 0.065 (0.018, 0.111) | 0.201 |
|  | Diastolic blood pressure | 0.007 (-0.018, 0.032) | 0.849 |
|  | Total physical activity | -0.002 (-0.027, 0.024) | 0.958 |
| UKB-weighted |  |  |  |
|  | Education | 1.00 (0.99, 1.00) | 0.849 |
|  | Weekly drinking | 1.00 (0.99, 1.01) | 0.948 |
|  | Current smoking | 1.00 (1.00, 1.01) | 0.797 |
|  | Family history of stroke | 1.00 (0.99, 1.00) | 0.877 |
|  | Systolic blood pressure | 0.056 (0.008, 0.103) | 0.246 |
|  | Diastolic blood pressure | 0.001 (-0.024, 0.027) | 0.959 |
|  | Total physical activity | -0.002 (-0.028, 0.024) | 0.958 |
| BBJ-BMI-weighted |  |  |  |
|  | Education | 1.00 (0.99, 1.00) | 0.797 |
|  | Weekly drinking | 1.00 (1.00, 1.01) | 0.849 |
|  | Current smoking | 1.00 (1.00, 1.01) | 0.849 |
|  | Family history of stroke | 1.00 (1.00, 1.01) | 0.797 |
|  | Systolic blood pressure | 0.029 (-0.007, 0.065) | 0.660 |
|  | Diastolic blood pressure | 0.025 (0.006, 0.045) | 0.201 |
|  | Total physical activity | -0.010 (-0.030, 0.009) | 0.797 |
| BBJ-BMI-unweighted |  |  |  |
|  | Education | 1.00 (0.99, 1.00) | 0.797 |
|  | Weekly drinking | 1.00 (1.00, 1.01) | 0.849 |
|  | Current smoking | 1.00 (1.00, 1.01) | 0.948 |
|  | Family history of stroke | 1.00 (1.00, 1.01) | 0.816 |
|  | Systolic blood pressure | 0.021 (-0.013, 0.056) | 0.797 |
|  | Diastolic blood pressure | 0.017 (-0.002, 0.036) | 0.489 |
|  | Total physical activity | -0.014 (-0.034, 0.005) | 0.660 |

**Notes:** GRS, genetic risk scores; UKB, UK Biobank; CKB, China Kadoorie Biobank; BBJ, Biobank Japan; BMI, body mass index. UKB-withoutBMI-uw, unweighted GRS of UKB without SNPs associated with BMI.

For the levels of blood pressure and total physical activity, the coefficient was *β*, and the linear regression models were applied. For the education (<9 years or ≥9 years), weekly drinking, current smoking, family history of stroke, and BMI (<24.0kg/m^2^ or ≥24.0kg/m^2^), the coefficient was odds ratio, and the logistic regression models were used. The models were adjusted for age, age^2^, sex, study areas, genetic array types, the first ten principal components. The P-values were adjusted for false discovery rate (FDR).

**Supplementary Table S5. Sensitivity analysis using two-stage method.**

| **Outcome** | **Analysis** | **GRS** | **HR (95%CI)** | **P** |
| --- | --- | --- | --- | --- |
| **Stroke** | | | | |
|  | All | UKB-weighted | 1.76 (1.30, 2.39) | <0.001 |
|  | All | UKB-withoutBMI-uw | 1.92 (1.35, 2.72) | <0.001 |
|  | Non-obese | UKB-weighted | 2.59 (1.58, 4.23) | <0.001 |
|  | Non-obese | UKB-withoutBMI-uw | 3.15 (1.75, 5.68) | <0.001 |
|  | MVMR | UKB-weighted | 2.01 (1.38, 2.93) | <0.001 |
|  | MVMR | UKB-withoutBMI-uw | 2.75 (1.71, 4.45) | <0.001 |
| **Hemorrhagic stroke** | | | | |
|  | All | UKB-weighted | 1.93 (1.08, 3.44) | 0.026 |
|  | All | UKB-withoutBMI-uw | 2.20 (1.13, 4.29) | 0.022 |
|  | Non-obese | UKB-weighted | 3.19 (1.34, 7.60) | 0.010 |
|  | Non-obese | UKB-withoutBMI-uw | 4.99 (1.75, 14.17) | 0.005 |
|  | MVMR | UKB-weighted | 2.71 (1.35, 5.45) | 0.006 |
|  | MVMR | UKB-withoutBMI-uw | 4.32 (1.78, 10.49) | 0.002 |
| **Ischemic stroke** | | | | |
|  | All | UKB-weighted | 1.71 (1.16, 2.54) | 0.008 |
|  | All | UKB-withoutBMI-uw | 2.22 (1.41, 3.48) | 0.002 |
|  | Non-obese | UKB-weighted | 2.70 (1.36, 5.34) | 0.006 |
|  | Non-obese | UKB-withoutBMI-uw | 4.34 (1.91, 9.89) | <0.001 |
|  | MVMR | UKB-weighted | 2.18 (1.33, 3.57) | 0.003 |
|  | MVMR | UKB-withoutBMI-uw | 4.19 (2.22, 7.91) | <0.001 |

**Notes:** GRS, genetic risk scores; UKB, UK Biobank. MVMR, multivariable MR. UKB-withoutBMI-uw, unweighted GRS of UKB without SNPs associated with body mass index (BMI).

HRs were scaled to represent the associations of a 0.5-fold increase in the prevalence of snoring and the average incident rate of outcomes. Both stages were adjusted for age, age^2^, sex, study areas, genetic array types, the first ten principal components, and BMI. Non-obese group included participants with BMI<24.0 kg/m^2^. For MVMR analysis, the first stage was additionally adjusted for the GRS of BMI, the second stage was additionally adjusted for the genetically-predicted BMI, and didn’t adjust for BMI in both stages. The P-values were adjusted for false discovery rate .

**Supplementary Table S6. SNPs for snoring identified in CKB GWAS and the replication in UKB GWAS.**

| **SNP** | **Chromosome** | **Position** | ***β*** | **SE** | **EA** | **NEA** | **MAF** | **P-value** | ***F*** | ***β*** **in UKB** | **SE in UKB** | **P in UKB** |
| --- | --- | --- | --- | --- | --- | --- | --- | --- | --- | --- | --- | --- |
| rs8043757 | 16 | 53813450 | -0.079597 | 0.0132755 | A | T | 0.122632 | 1.60E-09 | 35.95 | -0.00633916 | 0.00106863 | 3.00E-09 |
| rs712398 | 14 | 37385687 | 0.0503441 | 0.008867 | C | T | 0.405715 | 1.40E-08 | 32.24 | 0.00526592 | 0.00115444 | 5.30E-06 |
| rs2277339 | 12 | 57146069 | 0.0553677 | 0.0101845 | T | G | 0.239863 | 4.20E-08 | 29.56 | 0.00992786 | 0.00171425 | 5.90E-09 |

**Notes:** SNP, single-nucleotide polymorphisms; CKB, China Kadoorie Biobank; GWAS, genome-wide association study; SE, standard error; EA, effect allele; NEA, non-effect allele; MAF, minor allele frequency; UKB UK Biobank. GWAS of CKB was adjusted for age, age^2^, sex, study areas, genetic array types, the first ten principal components, and disease status at baseline. *F* statistic was calculated as (*β*/SE)^2^, the weak instrument bias was limited (*F*<10). The *β*, SE and P-value in UKB showed that the three SNPs all passed the replication in UKB.

**Supplementary Table S7. Tests for the MR analysis.**

| **Outcomes** | **No. of SNPs** | **Direction test** | | |  | **Pleiotropy test** | |  | **Heterogeneity test** | |
| --- | --- | --- | --- | --- | --- | --- | --- | --- | --- | --- |
|  |  | **r^2^.exposure** | **r^2^.outcome** | **Steiger P** |  | **Egger intercept (SE)** | **P** |  | **IVW Q** | **P** |
| **Stroke** | 3 | 0.0009802 | 2.25E-05 | TRUE P<0.001 | | 0.021 (0.047) | 0.7274481 |  | 0.2138512 | 0.8985925 |
|  | 2 | 0.0006185 | 2.02E-05 | TRUE P<0.001 | | NA | NA |  | 0.0307292 | 0.8608458 |
| **Hemorrhagic stroke** | 3 | 0.0009802 | 1.16E-05 | TRUE P<0.001 | | -0.001 (0.075) | 0.9902384 |  | 0.0197989 | 0.9901494 |
|  | 2 | 0.0006185 | 7.54E-06 | TRUE P<0.001 | | NA | NA |  | 0.0195375 | 0.8888364 |
| **Ischemic stroke** | 3 | 0.00098 | 3.83E-05 | TRUE P<0.001 | | 0.026 (0.055) | 0.719288 |  | 0.235 | 0.8891242 |
|  | 2 | 0.0006185 | 3.31E-05 | TRUE P<0.001 | | NA | NA |  | 5.15E-06 | 0.9981898 |

**Notes:** A direction test was performed using MR Steiger, comparing the variance of snoring explained by SNPs (r^2^.exposure) and variance of the outcomes explained by SNPs(r^2^.outcome). The intercept of MR-Egger was compared to zero to test for pleiotropy. Inverse-variance weighted (IVW) Cochrane’s Q test was used for the heterogeneity test.

**Supplementary Table S8. SNPs for snoring identified in UKB GWAS.**

| **SNP** | **Chromosome** | **Position** | **EA** | **NEA** | **P-value** | ***β*** | **SE** | **MAF** | **MAF_CKB** | **Info_CKB** | **P_HW__CKB** |
| --- | --- | --- | --- | --- | --- | --- | --- | --- | --- | --- | --- |
| rs725861 | 10 | 9063776 | A | G | 1.00E-11 | -0.009082 | 0.0013378 | 0.1918 | 0.226257 | 0.990037 | 0.0048936 |
| rs2049045 | 11 | 27694241 | G | C | 8.80E-10 | 0.0083411 | 0.0013415 | 0.1909 | 0.00148 | 1 | 1 |
| rs11018488^*^ | 11 | 88861590 | A | T | 5.30E-10 | 0.00678 | 0.001099 | 0.3638 | 0.00351 | 0.549842 | 0.343554 |
| 12:57088077_CA_C^*^ | 12 | 57088077 | CA | C | 3.00E-09 | 0.0068073 | 0.0011479 | 0.3052 | 0.216917 | 0.90719 | 0.992099 |
| rs10878269 | 12 | 65791463 | C | T | 2.30E-16 | -0.0088564 | 0.0010862 | 0.3499 | 0.390251 | 0.99843 | 0.0002456 |
| rs9583546 | 13 | 111566412 | G | C | 4.00E-08 | -0.0058679 | 0.0010805 | 0.3688 | 0.186743 | 0.997162 | 0.112024 |
| rs12429765 | 13 | 40745860 | A | G | 6.20E-11 | 0.0067995 | 0.0010508 | 0.493 | 0.437547 | 0.904562 | 2.42E-06 |
| rs592333 | 13 | 51340315 | A | G | 1.00E-17 | -0.0090579 | 0.0010512 | 0.4423 | 0.226441 | 1 | 0.276438 |
| rs2664299 | 14 | 99742187 | T | C | 1.10E-12 | 0.0075029 | 0.0010606 | 0.4145 | 0.065597 | 1 | 0.268929 |
| rs59502288 | 16 | 31109287 | G | GTCATCCA | 9.10E-10 | -0.0067292 | 0.0010942 | 0.3648 | 0.087776 | 0.998657 | 0.0489621 |
| rs796856741 | 16 | 53799278 | G | GT | 4.70E-11 | -0.0069611 | 0.0010592 | 0.4433 | 0.171493 | 0.990438 | 0.672547 |
| rs8069947 | 17 | 1985843 | C | T | 2.80E-10 | 0.006606 | 0.0010513 | 0.4881 | 0.283992 | 0.982136 | 0.0005763 |
| rs57222984 | 17 | 43758898 | A | G | 5.40E-12 | -0.0084345 | 0.0012201 | 0.2654 | 0.006564 | 0.92845 | 0.626493 |
| rs11409890^*^ | 17 | 46269542 | T | TA | 2.20E-10 | 0.006664 | 0.001061 | 0.4821 | 0.335814 | 0.934747 | 0.04389 |
| rs227727 | 17 | 54776955 | A | T | 1.40E-08 | -0.0059724 | 0.0010495 | 0.4503 | 0.322588 | 0.980106 | 0.0061199 |
| rs180107 | 17 | 67930772 | A | T | 2.10E-10 | -0.0067992 | 0.00106 | 0.3698 | 0.180993 | 0.985364 | 0.0299205 |
| rs9900496^*^ | 17 | 7439739 | T | C | 1.80E-09 | -0.00661 | 0.001106 | 0.3171 | 0.349463 | 0.91794 | 0.867961 |
| rs8108822 | 19 | 32183171 | C | T | 6.20E-10 | 0.0108729 | 0.0017827 | 0.1024 | 0.161808 | 1 | 0.323432 |
| rs80093081 | 1 | 39698437 | T | G | 1.80E-08 | 0.0063208 | 0.0011296 | 0.2922 | 0.35667 | 0.986133 | 0.234308 |
| 1:50819256_CA_C | 1 | 50819256 | CA | C | 3.50E-09 | 0.0062871 | 0.0010631 | 0.492 | 0.142326 | 0.917507 | 0.968066 |
| 1:87773720_CT_C | 1 | 87773720 | CT | C | 3.00E-08 | 0.0061027 | 0.0010967 | 0.338 | 0.227195 | 0.939107 | 0.0890984 |
| rs6099273 | 20 | 55347828 | C | T | 2.60E-08 | -0.006682 | 0.0012058 | 0.2346 | 0.19237 | 0.94415 | 0.353471 |
| rs6054427 | 20 | 6635266 | G | A | 4.00E-09 | -0.0063165 | 0.0010801 | 0.3777 | 0.132451 | 0.946793 | 0.857497 |
| rs61597598 | 2 | 156996626 | G | A | 5.10E-15 | -0.0118947 | 0.0015292 | 0.1163 | 0.025413 | 0.954248 | 0.94903 |
| rs74936745 | 3 | 77599166 | AC | A | 4.00E-10 | 0.0064901 | 0.0010503 | 0.4443 | 0.479585 | 0.997114 | 0.106598 |
| rs145367119^*^ | 3 | 90049321 | C | T | 1.20E-08 | 0.00604 | 0.001072 | 0.4314 | 0.432377 | 0.968018 | 0.048135 |
| rs202110996 | 3 | 94027330 | A | ATT | 4.60E-09 | 0.0062305 | 0.0010816 | 0.3936 | 0.365404 | 0.958776 | 0.0410024 |
| rs34732995 | 5 | 122699198 | C | CTA | 1.70E-08 | 0.0058234 | 0.0010479 | 0.4761 | 0.421589 | 0.991419 | 0.48957 |
| rs4976269 | 5 | 134452597 | G | A | 8.60E-10 | 0.0068439 | 0.0011141 | 0.341 | 0.191415 | 0.985344 | 0.0014201 |
| rs2307111 | 5 | 75003678 | T | C | 4.80E-13 | 0.0076674 | 0.0010698 | 0.3956 | 0.431285 | 1 | 0.792265 |
| rs17060460 | 6 | 100827834 | A | G | 1.40E-08 | -0.0070914 | 0.001247 | 0.2346 | 0.374003 | 0.996548 | 0.170588 |
| rs947612^*^ | 6 | 73738661 | G | A | 1.50E-08 | 0.006729 | 0.00121 | 0.2286 | 0.247612 | 1 | 2.37E-08 |
| rs2207944 | 6 | 84307328 | T | C | 2.00E-08 | -0.0059453 | 0.0010647 | 0.4573 | 0.382697 | 0.993566 | 0.0945673 |
| rs17151229 | 7 | 127382155 | G | C | 1.90E-09 | -0.0065399 | 0.0011024 | 0.3539 | 0.351413 | 0.968252 | 0.0002769 |
| rs13251292 | 8 | 71474355 | A | G | 4.30E-12 | -0.0073747 | 0.0010672 | 0.4145 | 0.067555 | 0.956791 | 0.312151 |
| rs7829639 | 8 | 78215352 | A | G | 1.40E-10 | -0.0074128 | 0.001155 | 0.2972 | 0.20168 | 0.979604 | 0.315394 |
| rs4744369 | 9 | 97475396 | T | A | 4.00E-08 | -0.0058253 | 0.001068 | 0.3956 | 0.2381 | 0.922231 | 0.359159 |

**Notes:** SNP, single-nucleotide polymorphisms; CKB, China Kadoorie Biobank; UKB, UK Biobank; GWAS, genome-wide association study; SE, standard error; EA, effect allele; NEA, non-effect allele; MAF, minor allele frequency; Info, information measure; P_HW_, P value for Hardy Weinberg equilibrium test. GWAS of UKB was adjusted for age, sex, genotyping array and the first 20 principal components in UKB.

^*^Six SNPs didn’t pass the quality control of CKB: rs11018488 with a low MAF and low Info in CKB; rs11409890 and rs57222984 (r^2^=0.003 in East Asian), rs180107 and rs9900496 (r^2^=0.001 in East Asian), rs145367119 and rs202110996 (r^2^=0.170, in East Asian), 12:57088077_CA_C and rs10878269 (r^2^=0.008 in East Asian) were in linkage disequilibrium (LD), rs947612 with a low P_HW_ in CKB. Four SNPs (rs12119849, rs773118143, rs34811474, rs4987719) were not found in CKB genotype data and were not shown in this table. There was no missing value of the dosage for each SNP.

**Supplementary Table S9. Genetic associations between snoring SNPs and BMI in CKB.**

| **GWAS** | **SNP** | **EA** | ***β*** | **SE** | **P-value** |
| --- | --- | --- | --- | --- | --- |
| UKB snoring GWAS | rs725861^†^ | G | -0.046 | 0.019 | 0.01841 |
|  | rs2049045 ^a^ | G | -0.347 | 0.209 | 0.09674 |
|  | rs10878269^†^ | T | -0.044 | 0.017 | 0.00797 |
|  | rs9583546 | C | 0.002 | 0.021 | 0.93019 |
|  | rs12429765 ^a^ | A | -0.018 | 0.017 | 0.30725 |
|  | rs592333 | G | 0.021 | 0.020 | 0.27909 |
|  | rs2664299^†^ | T | 0.073 | 0.033 | 0.02709 |
|  | rs59502288^†^ | GTCATCCA | 0.068 | 0.029 | 0.01721 |
|  | rs796856741^* a^ | GT | 0.253 | 0.021 | <1×10^-5^ |
|  | rs8069947^†^ | C | -0.051 | 0.018 | 0.00484 |
|  | rs57222984 | G | -0.015 | 0.098 | 0.87897 |
|  | rs227727 | T | 0.017 | 0.018 | 0.33683 |
|  | rs180107 | T | 0.020 | 0.021 | 0.35261 |
|  | rs8108822 | C | 0.032 | 0.022 | 0.15205 |
|  | rs80093081 | T | 0.009 | 0.017 | 0.60566 |
|  | rs6099273 | T | -0.002 | 0.021 | 0.9087 |
|  | rs6054427^†^ | A | 0.062 | 0.024 | 0.01054 |
|  | rs61597598 | A | -0.031 | 0.051 | 0.54528 |
|  | rs74936745 | AC | 0.022 | 0.016 | 0.17733 |
|  | rs202110996 | A | -0.002 | 0.017 | 0.90184 |
|  | rs34732995^*^ | C | 0.089 | 0.017 | <1×10^-5^ |
|  | rs4976269 ^a^ | G | -0.035 | 0.021 | 0.09089 |
|  | rs2307111^†^ | T | 0.074 | 0.016 | 0.00001 |
|  | rs17060460 | G | 0.017 | 0.017 | 0.31827 |
|  | rs2207944 | C | -0.004 | 0.017 | 0.8096 |
|  | rs17151229^†^ | C | 0.041 | 0.017 | 0.01649 |
|  | rs13251292 | G | 0.029 | 0.032 | 0.37485 |
|  | rs7829639 | G | 0.030 | 0.020 | 0.13546 |
|  | rs4744369 | A | -0.008 | 0.019 | 0.6774 |
|  | _1_50819256 | CA | 0.006 | 0.024 | 0.78899 |
|  | _1_87773720 | CT | -0.007 | 0.02 | 0.72376 |
| CKB snoring GWAS | rs2277339^†^ | T | 0.069 | 0.019 | 0.00026 |
|  | rs712398^†^ | C | -0.037 | 0.017 | 0.02522 |
|  | rs8043757^* a^ | T | 0.328 | 0.025 | <1×10^-5^ |

**Notes:** GRS, genetic risk scores; SNP, single-nucleotide polymorphisms; CKB, China Kadoorie Biobank; UKB, UK Biobank; GWAS, genome-wide association study; SE, standard error; EA, effect allele. BMI, body mass index. Linear regressions for SNPs and snoring were adjusted for age, age^2^, sex, study areas, genetic array types, and the first ten principal components.

^*^SNPs were excluded from the GRS construction for their highly associated with BMI (P < 1×10^-5^) in CKB population. ^†^SNPs were further excluded for their associations with BMI (P<0.05) in the sensitivity analysis. ^a^ SNPs were excluded for their associations with the level of blood pressure.

**Supplementary Table S10. SNPs for body mass index (BMI) identified in BBJ GWAS.**

| **SNP** | **Chromosome** | **Position** | **EA** | **NEA** | **P-value** | ***β*** | **SE** | **MAF** | **MAF_CKB** | **Info_CKB** | **P_HW__CKB** |
| --- | --- | --- | --- | --- | --- | --- | --- | --- | --- | --- | --- |
| rs491055 | 1 | 190308834 | G | A | 6.77E-09 | -0.022 | 0.004 | 0.63 | 0.365251 | 1 | 0.708571 |
| rs633715 | 1 | 177852580 | C | T | 2.72E-29 | 0.048 | 0.004 | 0.19 | 0.189552 | 1 | 0.021018 |
| rs860295 | 1 | 155767708 | G | A | 7.35E-10 | 0.029 | 0.005 | 0.28 | 0.266766 | 0.996768 | 0.498191 |
| rs9425762 | 1 | 173841352 | G | C | 4.18E-08 | 0.024 | 0.004 | 0.27 | 0.279879 | 0.989699 | 2.44E-05 |
| rs10192146^*^ | 2 | 198796423 | A | C | 3.62E-08 | 0.027 | 0.005 | 0.21 | 0.494265 | 0.707592 | 2.29E-68 |
| rs10174398^*^ | 2 | 51195601 | C | T | 1.16E-08 | -0.021 | 0.004 | 0.55 | 0.443211 | 0.953734 | 0.467813 |
| rs10197655^*^ | 2 | 58791420 | A | G | 2.15E-10 | -0.023 | 0.004 | 0.59 | 0.413785 | 0.991174 | 0.192951 |
| rs10208649 | 2 | 54161363 | C | T | 6.47E-11 | -0.109 | 0.017 | 0.01 | 0.005177 | 0.92326 | 1 |
| rs12617004 | 2 | 142615136 | C | G | 2.50E-08 | 0.02 | 0.004 | 0.32 | 0.317512 | 0.978641 | 0.146944 |
| rs2390669^†^ | 2 | 169091942 | C | A | 6.01E-10 | 0.025 | 0.004 | 0.32 | 0.330273 | 1 | 0.864261 |
| rs6734118 | 2 | 37559355 | A | C | 1.71E-10 | 0.023 | 0.004 | 0.59 | 0.348887 | 1 | 0.453821 |
| rs713586 | 2 | 25158008 | C | T | 4.61E-12 | 0.025 | 0.004 | 0.46 | 0.463101 | 1 | 0.689735 |
| rs77489951 | 2 | 38750287 | T | C | 2.56E-08 | 0.044 | 0.008 | 0.07 | 0.043185 | 0.993532 | 0.789256 |
| rs939584^†^ | 2 | 621558 | T | C | 1.39E-19 | 0.054 | 0.006 | 0.9 | 0.087374 | 0.999736 | 0.766951 |
| rs11130319 | 3 | 52755592 | T | A | 2.27E-10 | 0.023 | 0.004 | 0.51 | 0.438956 | 0.990488 | 0.179557 |
| rs4686392 | 3 | 185524081 | G | A | 1.87E-18 | -0.033 | 0.004 | 0.29 | 0.250274 | 0.997724 | 0.113832 |
| rs8192473 | 3 | 42299399 | T | C | 2.74E-08 | -0.034 | 0.006 | 0.11 | 0.117906 | 0.944962 | 0.327552 |
| rs1442493^*^ | 4 | 100321365 | A | G | 3.02E-08 | -0.025 | 0.004 | 0.67 | 0.342195 | 0.969724 | 0.622917 |
| rs1996023^†^ | 4 | 45164637 | G | T | 4.42E-16 | -0.032 | 0.004 | 0.67 | 0.323287 | 0.981301 | 0.227438 |
| rs10062657 | 5 | 95867908 | A | C | 5.69E-23 | -0.038 | 0.004 | 0.56 | 0.420635 | 0.925746 | 0.00923 |
| rs1035491 | 5 | 63962177 | G | A | 2.68E-09 | -0.026 | 0.004 | 0.25 | 0.284211 | 0.998544 | 0.520015 |
| rs1846974^*^ | 5 | 87969927 | A | G | 1.25E-13 | 0.027 | 0.004 | 0.53 | 0.497363 | 0.974955 | 0.156372 |
| rs4357030 | 5 | 124316031 | T | C | 1.64E-08 | 0.022 | 0.004 | 0.54 | 0.445542 | 0.966064 | 0.024134 |
| rs6881648 | 5 | 74991849 | C | A | 8.34E-12 | -0.024 | 0.004 | 0.53 | 0.432843 | 0.998212 | 0.887503 |
| rs148546399^*^ | 6 | 64705610 | A | G | 1.27E-08 | 0.048 | 0.009 | 0.02 | 0.000124 | 0.432116 | 1 |
| rs2206271 | 6 | 50786008 | A | T | 3.22E-16 | 0.031 | 0.004 | 0.33 | 0.342385 | 0.982304 | 0.623358 |
| rs183975233^*^ | 6 | 32437160 | A | T | 7.10E-15 | -0.031 | 0.004 | 0.33 | 0.34903 | 0.994856 | 0.000303 |
| rs35261542 | 6 | 20675792 | A | C | 9.92E-29 | -0.041 | 0.004 | 0.4 | 0.389576 | 0.98967 | 0.428066 |
| rs6913361 | 6 | 34179390 | G | A | 1.73E-12 | -0.037 | 0.005 | 0.87 | 0.097126 | 0.989214 | 0.437823 |
| rs9397585 | 6 | 153396875 | C | T | 3.39E-09 | 0.022 | 0.004 | 0.68 | 0.285573 | 0.978337 | 0.094336 |
| rs6947395 | 7 | 69406661 | T | A | 6.60E-12 | 0.031 | 0.004 | 0.19 | 0.213525 | 0.993891 | 0.947407 |
| rs28857569^†^ | 8 | 76697034 | C | T | 1.63E-09 | 0.024 | 0.004 | 0.24 | 0.214277 | 0.97797 | 0.856413 |
| rs4366055 | 8 | 95507328 | C | A | 2.23E-08 | -0.02 | 0.004 | 0.51 | 0.498014 | 0.991917 | 0.989893 |
| rs77636220^†^ | 8 | 64552779 | A | G | 7.85E-10 | 0.027 | 0.004 | 0.23 | 0.208653 | 0.926288 | 0.247293 |
| rs3932549 | 9 | 97073588 | C | A | 2.10E-08 | 0.024 | 0.004 | 0.65 | 0.367234 | 0.996005 | 0.978252 |
| rs5015933 | 9 | 128137418 | C | T | 3.66E-10 | -0.022 | 0.004 | 0.61 | 0.429152 | 0.985891 | 0.051013 |
| rs7020996 | 9 | 22129579 | T | C | 1.50E-19 | 0.035 | 0.004 | 0.41 | 0.416529 | 1 | 0.042438 |
| rs10795945 | 10 | 12302607 | C | T | 1.38E-09 | 0.022 | 0.004 | 0.48 | 0.429487 | 0.988768 | 0.886031 |
| rs1568079^†^ | 10 | 125251751 | A | T | 1.29E-11 | -0.025 | 0.004 | 0.37 | 0.331196 | 0.9833 | 0.407424 |
| rs1832886 | 10 | 94477539 | A | G | 4.63E-11 | 0.03 | 0.005 | 0.79 | 0.209821 | 0.990409 | 0.022267 |
| rs1907240 | 10 | 122897959 | A | G | 1.83E-11 | -0.026 | 0.004 | 0.64 | 0.397555 | 0.972405 | 0.331361 |
| rs2495707^*^ | 10 | 102425949 | G | A | 6.21E-09 | -0.025 | 0.004 | 0.43 | 0.441142 | 0.961341 | 0.53652 |
| rs4409766 | 10 | 104616663 | C | T | 2.16E-10 | 0.025 | 0.004 | 0.3 | 0.289525 | 0.997321 | 0.938765 |
| rs7903146^*^ | 10 | 114758349 | T | C | 3.85E-11 | -0.057 | 0.009 | 0.03 | 0.035081 | 0.986447 | 0.925714 |
| rs7912454^*^ | 10 | 18584792 | G | A | 2.02E-09 | -0.029 | 0.005 | 0.1 | 0.088227 | 1 | 0.844707 |
| rs11030100^†^ | 11 | 27677586 | T | G | 2.52E-26 | -0.038 | 0.004 | 0.47 | 0.467952 | 0.99035 | 0.893253 |
| rs11602339^†^ | 11 | 47761471 | T | C | 8.14E-10 | 0.024 | 0.004 | 0.33 | 0.309887 | 0.998684 | 0.888516 |
| rs16937956 | 11 | 8404501 | G | A | 9.65E-09 | -0.021 | 0.004 | 0.54 | 0.39153 | 0.987868 | 0.144632 |
| rs60808706 | 11 | 2857233 | A | G | 5.30E-36 | 0.046 | 0.004 | 0.38 | 0.339293 | 0.932224 | 0.746662 |
| rs7305242^†^ | 12 | 112256762 | C | T | 1.73E-08 | -0.022 | 0.004 | 0.55 | 0.377761 | 0.99312 | 0.045123 |
| rs80234489 | 12 | 31441179 | C | A | 1.88E-11 | -0.032 | 0.005 | 0.19 | 0.150251 | 0.960303 | 0.686333 |
| rs9568867^†^ | 13 | 54107352 | A | G | 1.52E-13 | 0.031 | 0.004 | 0.22 | 0.247726 | 1 | 0.138986 |
| rs729050^†^ | 14 | 94109502 | T | G | 1.91E-08 | 0.02 | 0.004 | 0.39 | 0.396161 | 0.945555 | 0.449491 |
| rs75766425 | 14 | 52511911 | C | G | 4.99E-13 | 0.04 | 0.006 | 0.12 | 0.117977 | 0.971621 | 0.028131 |
| rs72749754^†^ | 15 | 62319432 | C | G | 2.85E-08 | -0.024 | 0.004 | 0.21 | 0.21289 | 0.981237 | 0.407938 |
| rs11642015^†^ | 16 | 53802494 | T | C | 4.91E-72 | 0.079 | 0.004 | 0.16 | 0.123897 | 0.991369 | 0.941959 |
| rs12597682 | 16 | 20258432 | A | C | 6.80E-11 | -0.03 | 0.005 | 0.24 | 0.267971 | 0.997735 | 0.82178 |
| rs2540034 | 16 | 4022694 | T | C | 3.49E-11 | 0.028 | 0.004 | 0.32 | 0.324056 | 0.900324 | 0.009981 |
| rs62034325^*^ | 16 | 28538640 | G | A | 1.70E-10 | 0.035 | 0.005 | 0.1 | 0.096907 | 0.990419 | 1.69E-06 |
| rs4790981 | 17 | 65921834 | G | A | 7.69E-09 | 0.023 | 0.004 | 0.66 | 0.375134 | 0.993403 | 5.98E-06 |
| rs55934576 | 17 | 45725552 | C | T | 2.22E-08 | -0.024 | 0.004 | 0.29 | 0.256075 | 0.988398 | 0.204645 |
| rs1518170 | 18 | 40708905 | C | T | 2.37E-08 | -0.021 | 0.004 | 0.57 | 0.430959 | 0.992278 | 0.005761 |
| rs6567160 | 18 | 57829135 | C | T | 1.52E-32 | 0.051 | 0.004 | 0.2 | 0.203532 | 0.998927 | 0.015855 |
| rs35560038 | 19 | 46175046 | T | A | 6.53E-50 | -0.055 | 0.004 | 0.53 | 0.496613 | 0.984466 | 0.341155 |
| rs16978956 | 20 | 18288165 | G | A | 6.58E-09 | 0.026 | 0.005 | 0.19 | 0.139082 | 0.989998 | 0.989452 |
| rs4811309 | 20 | 50817740 | A | G | 3.48E-08 | -0.021 | 0.004 | 0.35 | 0.323498 | 0.969712 | 0.695424 |
| rs6089584^*^ | 20 | 60564086 | C | G | 4.19E-08 | -0.021 | 0.004 | 0.65 | 0.340278 | 0.983773 | 0.749253 |
| rs139913^†^ | 22 | 40713861 | A | T | 2.45E-13 | -0.026 | 0.004 | 0.47 | 0.424518 | 0.983309 | 0.521111 |

**Notes:** SNP, single-nucleotide polymorphisms; CKB, China Kadoorie Biobank; BBJ, Biobank Japan; GWAS, genome-wide association study; SE, standard error; EA, effect allele; NEA, non-effect allele; MAF, minor allele frequency; Info, information measure; P_HW_, P value for Hardy Weinberg equilibrium test. GWAS of UKB was adjusted for age, age^2^, sex, disease status at baseline and the first ten principal components in BBJ.

^*^12 SNPs didn’t pass the quality control of CKB: rs148546399 with a low MAF and low Info in CKB, rs10192146 with a low P_HW_ in CKB; rs10174398 and rs10208649 (r^2^=0.002 in East Asian, the same below), rs10197655 and rs10208649 (r^2^=0.009), rs1846974 and rs10062657 (r^2^=0.002), rs2495707 and rs1832886 (r^2^=0.002), rs7903146 and rs1907240 (r^2^=0.002), rs7912454 and rs10795945 (r^2^=0.002), rs12597682 and rs62034325 (r^2^=0.008), rs6089584 and rs4811309 (r^2^=0.003) , rs1442493 and rs1996023 (r^2^=0.002) were in linkage disequilibrium (LD). rs183975233 was on the major histocompatibility complex regions and was not found in 1000G EAS genotype data. There was no missing value of the dosage for each SNP. ^†^rs11642015 was dropped for it’s in LD with the snoring SNP rs8043757 [r^2^=0.972]. rs7305242 was dropped for its highly association with alcohol drinking in CKB. rs939584, rs72749754, rs1996023, rs28857569, rs1568079, rs11602339 were dropped for their associations with the level of blood pressure.
