# Supplementary figures and images for: Causal association between snoring and stroke: a Mendelian randomization study in a Chinese population
Source: Lancet Reg Health West Pac. 2024 Jan 23;44:101001. doi: 10.1016/j.lanwpc.2023.101001 (PMC10832459; doi:10.1016/j.lanwpc.2023.101001)

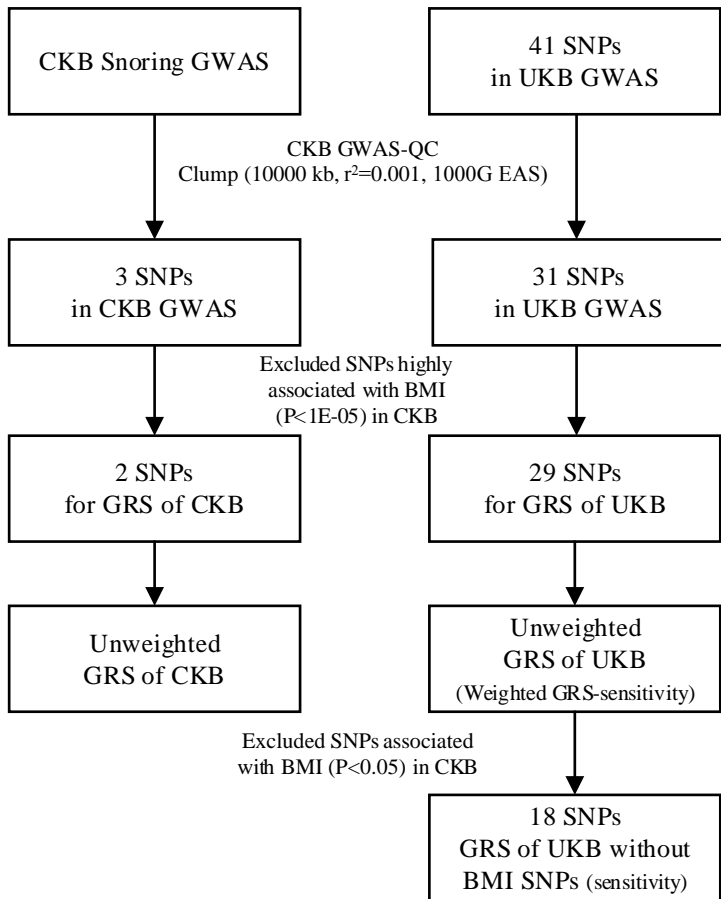

Supplement: Supplementary Figure S1 [file mmc3.pdf]
