# Supplementary material for: Causal association between snoring and stroke: a Mendelian randomization study in a Chinese population
Source: Lancet Reg Health West Pac. 2024 Jan 23;44:101001. doi: 10.1016/j.lanwpc.2023.101001 (PMC10832459; doi:10.1016/j.lanwpc.2023.101001)
Supplement: Supplementary Figure S2 [file mmc4.pdf]

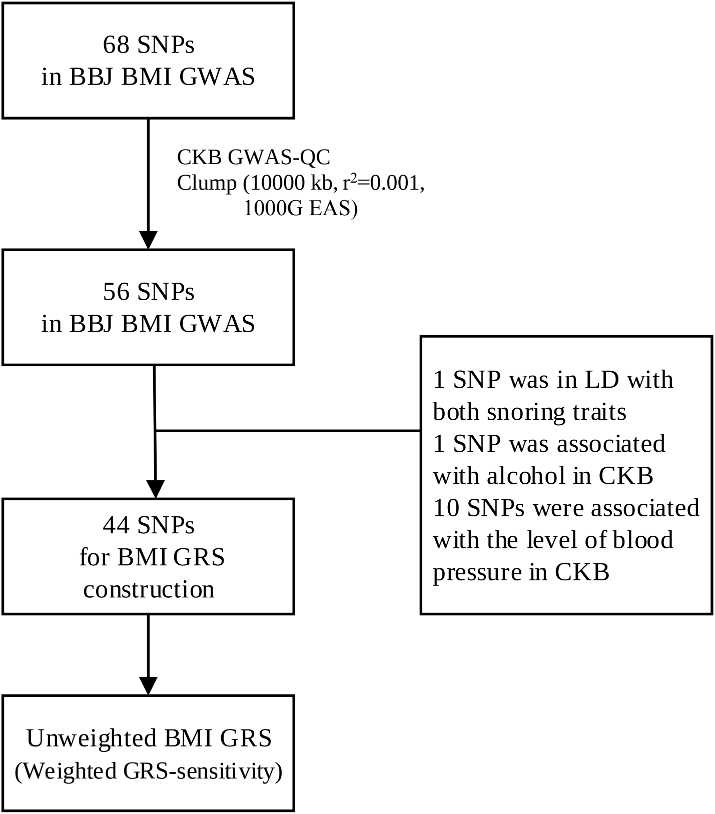
68 SNPs
in BBJ BMI GWAS
CKB GWAS-QC
Clump (10000 kb, r2=0.001, 1000G EAS)
56 SNPs
in BBJ BMI GWAS
1 SNP was in LD with both snoring traits
1 SNP was associated with alcohol in CKB
10 SNPs were associated with the level of blood pressure in CKB
44 SNPs
for BMI GRS
construction
Unweighted BMI GRS
(Weighted GRS-sensitivity)
