# Supplementary material for: Causal association between snoring and stroke: a Mendelian randomization study in a Chinese population
Source: Lancet Reg Health West Pac. 2024 Jan 23;44:101001. doi: 10.1016/j.lanwpc.2023.101001 (PMC10832459; doi:10.1016/j.lanwpc.2023.101001)
Supplement: Translated Abstract [file mmc5.docx]

*This translation in Chinese was submitted by the authors and we reproduce it as supplied. It has not been peer reviewed. Our editorial processes have only been applied to the original abstract in English, which should serve as reference for this manuscript.*

**摘要**

**背景：**既往观察性研究表明，打鼾和脑卒中之间存在正向关系。本研究旨在探究打鼾对脑卒中的因果效应。

**方法：**基于中国慢性病前瞻性队列研究（China Kadoorie Biobank，CKB）中82,339名无亲缘关系亚洲人的经过质量控制的遗传数据和表型数据，本研究使用孟德尔随机化（Mendelian randomization，MR）的方法，对打鼾与脑卒中的因果关联进行分析。本研究使用在CKB与英国生物银行（UK Biobank，UKB）中打鼾全基因组关联研究发现的打鼾易感基因位点来构建基因风险评分（genetic risk scores，GRS），使用两阶段分析法来衡量遗传预测的打鼾可能性与脑卒中及其亚型之间关联。为控制体质指数（body mass index, BMI）可能产生的多效性偏倚，额外在非肥胖组（BMI<24 kg/m^2^）进行MR分析，并进行多变量MR分析。此外，本研究基于CKB中打鼾易感基因位点，使用了逆方差加权法衡量二者关联。

**结果：**MR结果显示，打鼾与脑卒中、出血性脑卒中、缺血性脑卒中有显著的正向关联，使用基于CKB的打鼾易感基因位点构建的GRS相应的HR（95%CI）为1.56（1.15，2.12）、1.50（0.84，2.69）、2.02（1.36，3.01），使用基于UKB的打鼾易感基因位点构建的GRS相应的HR（95%CI）为1.78（1.30，2.43）、1.94（1.07，3.52）、1.74（1.16，2.61）。在非超重肥胖组、多变量MR、使用逆方差加权法的MR分析中，打鼾与脑卒中及各亚型之间的关联性保持稳定。

**解释：**本研究结果表明，在中国成人中，遗传预测的打鼾可能性较高，与脑卒中、出血性脑卒中、缺血性脑卒中的发病风险升高存在正向关联，该关联独立于BMI的作用。
